# Metabolomics dissection of depression heterogeneity and related cardiometabolic risk

**DOI:** 10.1017/S0033291721001471

**Published:** 2023-01

**Authors:** Tahani Alshehri, Dennis O. Mook- Kanamori, Ko Willems van Dijk, Richard Dinga, Brenda W. J. H. Penninx, Frits R. Rosendaal, Saskia le Cessie, Yuri Milaneschi

**Affiliations:** 1 Department of Clinical Epidemiology, Leiden University Medical Center, Leiden, The Netherlands; 2 Department of Public Health and Primary Care, Leiden University Medical Center, Leiden, The Netherlands; 3 Department of Human Genetics, Leiden University Medical Center, Leiden, The Netherlands; 4 Department of Internal Medicine, Division of Endocrinology, Leiden University Medical Center, Leiden, The Netherlands; 5 Donders Institute for Brain, Cognition and Behaviour, Radboud University, Nijmegen, The Netherlands; 6 Department of Psychiatry, Amsterdam Public Health Research Institute, Amsterdam Neuroscience, Amsterdam UMC, Vrije Universiteit, The Netherlands; 7 Department of Biomedical Data Sciences, Leiden University Medical Center, Leiden, The Netherlands; 8 GGZ inGeest, Research & Innovation, Amsterdam, The Netherlands

**Keywords:** Body fat distribution, body mass index, depression, metabolic syndrome, metabolomics

## Abstract

**Background:**

A recent hypothesis postulates the existence of an ‘immune-metabolic depression’ (IMD) dimension characterized by metabolic dysregulations. Combining data on metabolomics and depressive symptoms, we aimed to identify depressions associated with an increased risk of adverse metabolic alterations.

**Method:**

Clustering data were from 1094 individuals with major depressive disorder in the last 6 months and measures of 149 metabolites from a ^1^H-NMR platform and 30 depressive symptoms (IDS-SR30). Canonical correlation analyses (CCA) were used to identify main independent metabolite-symptom axes of variance. Then, for the replication, we examined the association of the identified dimensions with metabolites from the same platform and cardiometabolic diseases in an independent population-based cohort (*n* = 6572).

**Results:**

CCA identified an overall depression dimension and a dimension resembling IMD, in which symptoms such as sleeping too much, increased appetite, and low energy level had higher relative loading. In the independent sample, the overall depression dimension was associated with lower cardiometabolic risk, such as (i.e. per s.d.) HOMA-1B −0.06 (95% CI −0.09 – −0.04), and visceral adipose tissue −0.10 cm^2^ (95% CI −0.14 – −0.07). In contrast, the IMD dimension was associated with well-known cardiometabolic diseases such as higher visceral adipose tissue 0.08 cm^2^ (95% CI 0.04–0.12), HOMA-1B 0.06 (95% CI 0.04–0.09), and lower HDL-cholesterol levels −0.03 mmol/L (95% CI −0.05 – −0.01).

**Conclusions:**

Combining metabolomics and clinical symptoms we identified a replicable depression dimension associated with adverse metabolic alterations, in line with the IMD hypothesis. Patients with IMD may be at higher cardiometabolic risk and may benefit from specific treatment targeting underlying metabolic dysregulations.

## Introduction

Cardiovascular disease (CVD) together with major depressive disorder (MDD) are leading causes of mortality and disease burden worldwide (Dhar & Barton, 2016; Mathers & Loncar, 2006). Each of these conditions may predispose for the other, and the presence of one condition worsens the prognosis of the other (Penninx, Milaneschi, Lamers, & Vogelzangs, 2013). Although the mechanism of this comorbidity is still not fully understood, adverse metabolic alterations may serve as the element that connects the two conditions (Dhar & Barton, 2016; Khandaker et al., 2020; Penninx et al., 2013). A recent large-scale epidemiological study in > 15 000 individuals analyzing the association between depression and more than 200 lipid-related metabolites (Bot et al., 2020) found that depression is associated with a metabolic signature that is also found in CVD patients (Holmes et al., 2018). This metabolic signature was characterized by a shift in the lipids levels encompassing less HDL-cholesterol and more very low density lipoproteins (VLDL) and triglycerides, in line with a higher metabolic syndrome profile in depression (Bot et al., 2020). This metabolic signature may represent a substrate linking depression to cardiometabolic diseases. Another large population-based study in > 350 000 individuals (Khandaker et al., 2020) concluded that the risk factors of CVD [i.e. inflammatory markers (CRP, IL-6) and biomarker (triglycerides)] are likely causal for the development of depression.

MDD is a highly heterogeneous disorder: patients with the same MDD diagnoses according to DSM-V (Diagnostic and Statistical Manual of Mental Disorders) (American Psychiatric Association, 2013) may experience very different symptom profiles (Lux & Kendler, 2010). These different clinical expressions may be, in turn, differentially related to underlying biological dysregulations. Recent evidence suggests that adverse metabolic alterations and inflammatory dysregulation map more consistently onto ‘atypical, energy-related depressive symptoms,’ such as excessive sleepiness, hyperphagia, weight gain, and fatigue (Milaneschi, Lamers, Berk, & Penninx, 2020). This set of symptoms is partially shared with other constructs, such as sickness behavior (Miller & Raison, 2016) and nosological categories, such as atypical depression, seasonal affective disorder, and bipolar disorder (American Psychiatric Association, 2013). The clustering of atypical, energy-related depressive symptoms with inflammatory and metabolic alterations indexes an underlying quantitative dimension, labelled ‘immuno-metabolic depression’ (IMD), with transdiagnostic value and potentially present in psychiatric (depression, bipolar or psychotic disorders) and somatic (obesity, diabetes, cardiovascular) disorders characterized by overlapping symptomatology or biological dysregulations (Milaneschi et al., 2020). Nonetheless, further empirical evidence is needed to fully characterize the clustering between specific symptom profiles and immuno-metabolic biological dysregulations. The identification of depression dimensions characterized by this clustering of clinical and biological features could give us a better understanding of the shared biological mechanisms between depression and cardiometabolic diseases and potential opening for interventions aimed at avoiding their reciprocal influence (Baune et al., 2012; Fried & Nesse, 2015; Lamers, Milaneschi, de Jonge, Giltay, & Penninx, 2018). Furthermore, the identification of individuals with this specific form of depression may create awareness amongst healthcare providers and the need to perform more rigorous cardiometabolic health checks and interventions.

The main aim of the present study was to identify depression dimensions associated with increased risk of adverse metabolic profile by combining data on metabolomics and depressive symptoms. First, we applied a data-driven method to identify patterns of correlations between depressive symptoms and metabolites from a lipid-focused metabolomic platform in >1000 MDD patients. Previous studies aimed at parsing depression heterogeneity through data-driven methods followed two conceptually distinct approach (online Supplementary Fig. S1 adapted from Buch & Liston, 2021). In one approach (top-down), studies (Chu et al., 2019; Lamers et al., 2013) performed symptom-based clustering as a first step and subsequently evaluated the clustering results via association with biomarker levels. In the opposite approach (bottom-up), studies (Beijers et al., 2019; Osimo et al., 2020) performed biomarker-based clustering as a first step and subsequently evaluated the clustering results via association with clinical features. The novelty of the present study is that we merged the two approaches and performed clustering based on both symptoms and biomarkers, leveraging their co-variance structure. Then, for the replication, we examined the association between the identified dimensions and 51 metabolites from the same panel, and clinical cardiometabolic diseases such as levels of fasting glucose, insulin resistance, total and abdominal adiposity in an independent population-based cohort (*n* = 6572).

## Method

### Study design

**Fig. 1.**
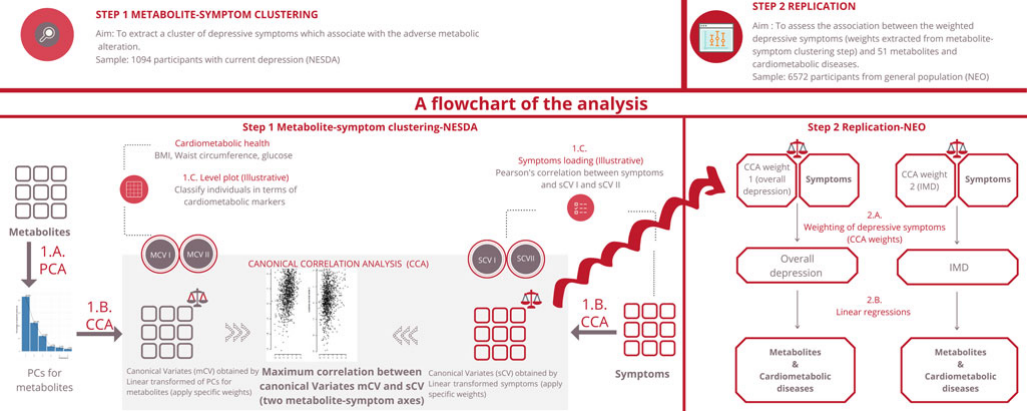
An illustration of the method.

The current analysis consists of two parts: the metabolite-symptom clustering and the replication (Fig. 1). In the first part, we used a data-driven approach to dissect the heterogeneity of depression and to identify main independent metabolite-symptom dimension of variance in 1094 individuals with depression in the last 6 months from the Netherlands Study of Depression and Anxiety cohort (NESDA). Then, in the replication, we examined the association between the dimensions identified and the cardiometabolic metabolites (51 lipids, fatty acids, and low-molecular-weight metabolites) and diseases in an independent dataset of 6572 participants from the general population enrolled in the Netherlands Epidemiology of Obesity (NEO) study. The research protocol of NESDA was approved by the medical ethical committees of the following participating universities: Leiden University Medical Center (LUMC), Vrije University Medical Center (VUMC), and University Medical Center Groningen (UMCG). The NEO study was approved by medical ethics committee of Leiden University Medical Center (LUMC). All participants gave written informed consent.

### Part 1: Metabolite-symptom clustering

We performed this analysis on 1094 participants diagnosed with MDD in the last 6 months via the structured Composite Interview Diagnostic Instrument (CIDI, version 2.1) (Robins et al., 1988) from NESDA (Penninx et al., 2008). After an overnight fast, EDTA plasma was collected and stored in aliquots at −80 °C until further analysis by ^1^H-NMR Nightingale Health Ltd, Helsinki, Finland (Soininen, Kangas, Würtz, Suna, & Ala-Korpela, 2015) metabolomics platform. This metabolomics platform consists of 230 metabolites or metabolite ratios and can be classified into three clusters (Würtz et al., 2017) as follows: (1) lipids, fatty acids, and low-molecular-weight metabolites (*n* = 51); (2) lipid composition and particle concentration measures of lipoprotein subclasses (*n* = 98); and (3) metabolite ratios (*n* = 81). In this analysis, we focused on the first two classes (*n* = 149). Metabolite ratios were not used due to redundancy. We processed the metabolomic data based on the protocol described in online Supplementary Appendix 1 that was suggested by the manufacturer of the platform and has been consistently applied in several large-scale epidemiological studies (Bot et al., 2020; Onderwater et al., 2019). Blood samples were analyzed in two batches (April 2014 and December 2014) by ^1^H-NMR Nightingale Health Ltd, Helsinki, Finland) (Soininen et al., 2015). We regressed the metabolites on age and batch effect in order to remove their confounding effect.

During the baseline assessment, the presence of MDD was determined with the Diagnostic and Statistical Manual of Mental Disorders, Fourth Edition (DSM-IV)-based Composite Interview Diagnostic Instrument (CIDI, version 2.1, World Health Organization, 1997) by specially trained research staff. Additionally, participants were asked to complete the Inventory of Depressive Symptomatology (IDS-SR30), which assesses (via a 4-level response system) the presence of 30 depressive symptoms during the last week and their severity (Rush, Gullion, Basco, Jarrett, & Trivedi, 1996). Additional measures of body mass index (BMI), waist circumference and fasting glucose level are described in detail in online Supplementary Appendix 2.

#### Statistical analysis for metabolite-symptom clustering

Our goal was to identify independent dimensions emerging from patterns of correlations between depressive symptoms and metabolites. For that, we used canonical correlation analysis (CCA, Hotelling, 1936).

1.A. Principal component analysis (PCA): Metabolites are correlated to each other; to avoid overfitting and unstable results of CCA, data reduction (Dinga et al., 2019) of metabolomics was performed applying PCA to age- and batch-adjusted metabolites residuals. PCA is described in more detail in online Supplementary Appendix 3. We selected principal components explaining the highest proportion of variance (components that explained more than 10% of variance) in metabolites. Therefore, the next analysis was performed on principal components explaining the highest proportion of metabolites variance and 30 depressive symptoms.

1.B. Canonical correlation analysis (CCA): CCA (Hotelling, 1936) is a method that given two sets of variables X and Y (in this case, metabolites and depressive symptoms), find a linear combination of X that is maximally correlated with a linear combination of Y (i.e. a weighted sum of each variable). Detailed definition and description of CCA method explained in online Supplementary Appendix 4. In our analysis we chose to proceed with the first two canonical pairs that provided more information about the two sets of variables. The relationship between the created canonical variables of depressive symptoms and metabolites from the same panel and cardiometabolic diseases was validated in an independent sample (see replication section).

1.C. Illustrative analyses: In order to better explain the results of CCA and the meaning of its output we proposed two additional analyses (point 1.C in Fig. 1). To explore how the first two metabolic canonical variates (mCVI and mCVII) classify individuals in terms of cardiometabolic diseases (i.e. BMI, waist circumference, fasting glucose) we plotted the predicted level of the cardiometabolic diseases as a function of the two metabolic canonical variates (i.e. smoothing function was used for the prediction). Furthermore, to evaluate the symptoms contribution to the two canonical correlation, for each symptom we calculated the symptoms loadings, expressed in Pearson's correlation coefficient, with the first two symptoms canonical variates (sCVI and sCVII).

### Part 2: Replication

To replicate the results of the previous step, we investigated the association between the dimensions identified in the previous step via CCA and metabolomics and cardiometabolic diseases in the Netherlands Epidemiology of Obesity (NEO) study (de Mutsert et al., 2013). The depressive symptoms in NEO study were assessed by IDS-SR30 (Rush et al., 1996), the same instrument used in the NESDA study. For the purpose of replication, we included only the first class from the ^1^H-NMR platform (i.e. 51 lipids, fatty acids, and low-molecular-weight metabolites) in the main results. For completeness of data, we showed the result of the entire metabolomic platform in the supplementary results since they have large overlap with the standard clinical lipid profile. We used the same protocol for processing this metabolomic data in the clustering step. The cardiometabolic diseases are described in detail elsewhere (de Mutsert et al., 2013). From fasting glucose and insulin concentrations, we calculated the Homeostasis Model Assessment for Insulin Resistance (HOMA-IR) and HOMA of beta-cell function (HOMA-1B) as markers of hepatic insulin resistance and steady-state insulin secretion (Matthews et al., 1985). HOMA-IR was calculated as fasting insulin (*μ*U/mL) × fasting glucose (mmol/L)/22.5 and HOMA-1B% as 20 × fasting glucose (mmol/l)-3.5 (Matthews et al., 1985; Wallace, Levy, & Matthews, 2004).

#### Statistical analysis for replication

2.A. Weighting of depressive symptoms: To index the two dimensions identified in the clustering step, we created two weighted depressive symptom scores. We weighted each individual item of the IDS-SR30 based on extracted CCA weights from the previous step. Then, we summed the weighted depressive symptoms to create two weighted IDS scores. We standardized weighted IDS scores to a mean of zero and a standard deviation of one to allow comparison across the scores.

2.B. Linear regressions: We used linear regression to examine the relationship between the two weighted IDS scores as the independent variable and 51 ^1^H-NMR metabolites and cardiometabolic diseases (BMI, total body fat, waist circumference, visceral adipose tissue, HbA1c, fasting glucose, HOMA-IR, HOMA-1B, total cholesterol, LDL-cholesterol, HDL-cholesterol, and triglycerides) as dependent variables. We fitted four linear regression models, the crude model, model 1, model 2, and model 3. Model 1 was adjusted for age, sex, and educational level. Model 2 was adjusted for age, sex, educational level, smoking, alcohol consumption, physical activity, and ethnicity. Model 3 was model 2 with additional adjustment for lipid-lowering drugs, and antidepressants. The false discovery rate (FDR) method was applied to correct for the multiple testing. As the NEO study is a population-based study with oversampling of individuals with a BMI > 27 kg/m^2^, all results are based on BMI-weighted analysis. The weighting factor is based on BMI distribution in the general Dutch population to make our results generalizable to the Dutch population.

## Results

### Part 1: Metabolite-symptom clustering

Table 1 shows the main demographic, health- and depression-related characteristic, in the NESDA sample of individuals with MDD in the last 6 months.

**Table 1.** Characteristics of the study population for the metabolite-symptom clustering step (NESDA)

	Metabolite-symptom clustering (NESDA *n* = 1094)
*N*	1094
Women, *n* (%)	741 (67.73)
Age (years) (mean, s.d.)	40.88 (12.11)
High educational level (high) *n* (%)	306 (27.97)
Use of lipid-modifying medications, (yes) *n* (%)	78 (7.13)
BMI (kg/m^2^) (mean, s.d.)	25.90 (5.51)
Waist circumference (cm)	89.58 (14.56)
Glucose (mmol/L)	5.20 (1.15)
Use of antidepressant (Yes) *n* (%)	477 (43.60)
Total IDS-score (0–84) (mean, s.d.)	32.5 (12.2)

IDS-SR30, Inventory of Depressive Symptomatology (self-report); BMI, body mass index; NESDA, Netherlands study for depression and anxiety.

Normally distributed data shown as mean and standard deviation (s.d.), skewed distributed data shown as median (25th, 75th percentiles), and categorical data are shown as percentage. High education level: university or college education, while other education level: none, primary school, or lower vocational education.

#### 1.A. Principal component analysis

Data reduction of metabolomics was performed using PCA, identifying three principal components that explained more than 10% of the variance in metabolites (together explained 75% of the variance) (Scree plot in online Supplementary Fig. S2).

#### 1.B. Canonical correlation analysis

The resulting three principal components were used in the CCA analysis and were correlated to the 30 depressive symptoms, to identify the main independent metabolite-symptom dimensions of variance based on their correlation. The correlation between the linear transformation (weights) of metabolites principal components (metabolic canonical variate I, mCVI) and depressive symptoms (symptom canonical variate I, sCVI) was 0.30 explaining 54% of the metabolite-symptom covariance, for the second pair of canonical variates the correlation between mCVII and sCVII was 0.24 explaining 33% of the metabolite-symptom covariance (online Supplementary Fig. S3).

#### 1.C. Illustrative analyses

**Fig. 2.**
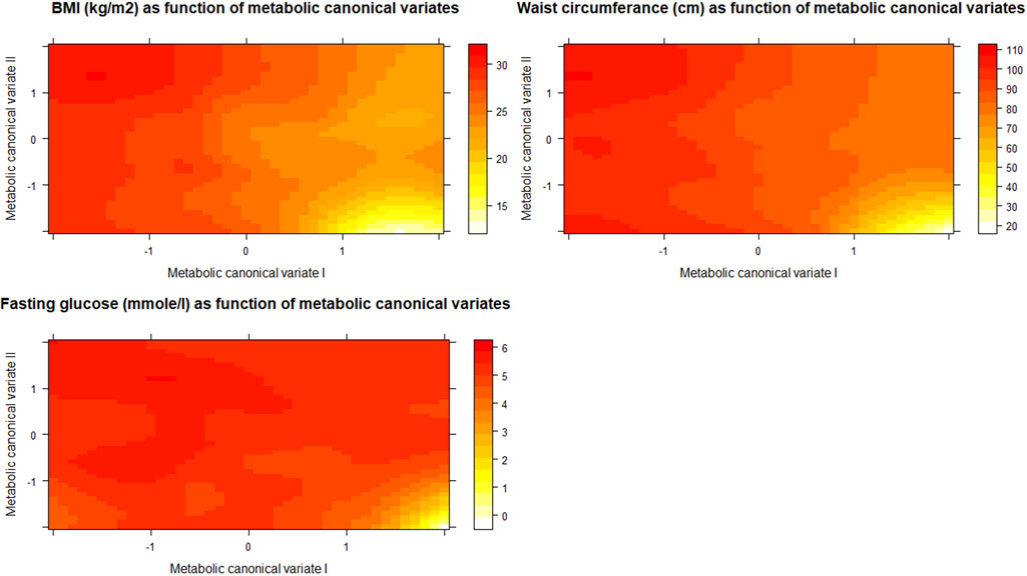
Level plots of the predicted cardiometabolic diseases as functions of the first and second metabolic canonical variates. sCV I: First symptoms canonical variates I. sCV II: Second symptoms canonical variates.

**Fig. 3.**
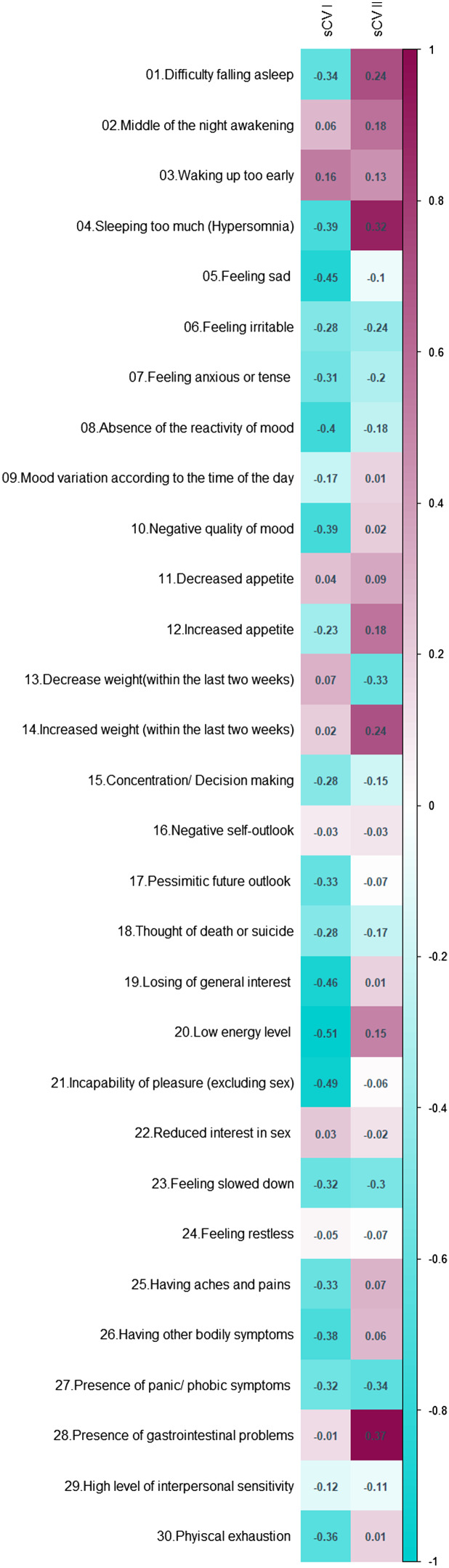
Canonical loading of depressive symptoms on the symptoms canonical variates.

To explore how the first two metabolic canonical variates (mCVI and mCVII) classify individuals in terms of cardiometabolic diseases (i.e. measures of BMI, waist circumference, fasting glucose) we plotted the predicted level of the diseases as a function of the two metabolic canonical variates. Level plots depicted in Fig. 2 show that high values in BMI, waist circumference, and fasting glucose tended to cluster at high levels of mCVII and low levels for mCVI. Figure 3 shows the loading, expressed as Pearson's correlation coefficient, of IDS-SR item on the two symptoms canonical variates (sCVI and sCVII). In the first variate, correlation coefficients were substantially consistent across the entire spectrum of items, including mood, cognitive and somatic symptoms. In the second variate, the loading of specific items such as difficulty falling asleep, sleeping too much, increase weight and appetite, low energy level and gastrointestinal problems were relatively higher as compared to the other symptoms.

We interpreted the first canonical variate CVI, explaining a larger proportion of symptom-metabolite covariance (54%), as an overall depression dimension characterized by a wide array of symptoms (sCVI, Fig. 3) and lower levels of cardiometabolic diseases (mCVI, Fig. 2). The second variate, explaining 33% of the symptom-metabolite covariance, partially resembled the postulated IMD construct (Milaneschi et al., 2020), with relevance for energy-related behavioral symptoms and higher cardiometabolic diseases. Thus, for interpretability we labelled the two canonical variates, respectively, ‘overall depression’ and ‘IMD’.

### Part 2: Replication

The baseline characteristics for all 6572 participants of the NEO cohort included in the replication step are shown in online Supplementary Table S1. The mean age in the NEO population was 55.7 years [standard deviation (s.d.)]: 6 years, and the median of the IDS-SR30 questionnaire was 8.0 points (4, 13).

#### 2.A. Weighting of depressive symptoms

We created two weighted depressive symptoms scores labelled ‘overall depression’ and ‘IMD’ with the weights derived in CCA for, respectively, the first and second canonical variate.

#### 2.B. Linear regression

**Fig. 4.**
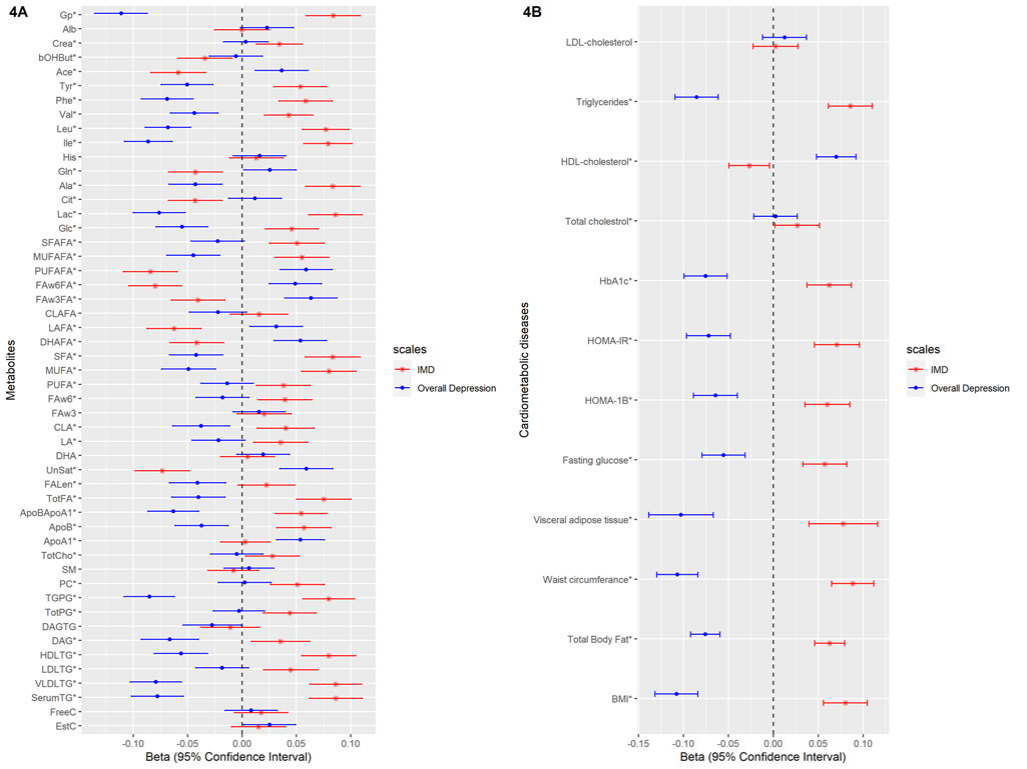
The linear regression analysis of the association between the weighted depressive symptoms scores and the cardiometabolic diseases and metabolites in individuals from NEO study. The weights extracted from the metabolite-symptom clustering step. * FDR significant (*q* < 0.05) at least for one of the two depressive symptom scales. Number of individuals with data for BMI: 6572, total body fat: 6541, waist circumference: 6566, visceral adipose tissue: 2537, fasting glucose: 6554, HOMA-1B: 6541, HOMA-IR: 6545, HbA1c: 6543, total cholesterol: 6562, HDL-cholesterol: 6561, triglycerides: 6561, LDL-cholesterol: 6560.

We examined the association of these weighted scores with 51 metabolites and cardiometabolic diseases (levels of BMI, total body fat, waist circumference, visceral adipose tissue, HbA1c, fasting glucose, HOMA-IR, HOMA-1B, total cholesterol, LDL-cholesterol, HDL-cholesterol and triglycerides). Figures 4a and 4b depict the linear regression effect estimates and 95% confidence intervals for the association between the weighted symptom sum score and the 51 lipids, fatty acids, and low-molecular-weight metabolites, and cardiometabolic diseases adjusted for age, sex, and educational level (model 1). The results of all crude and adjusted models can be found in online Supplementary Tables S2 and S3. In general, the two weighted symptoms scores showed divergent pattern of results: IMD showed metabolic alterations linked to increased cardiometabolic risk, while overall depression score showed opposite associations. IMD was associated with [per standard deviation (s.d.)] higher glycoprotein acetylase 0.08 mmol/L (95% CI 0.06–0.11), apolipoprotein B 0.06 g/L (95% CI 0.03–0.08), triglyceride levels 0.09 mmol/L (95% CI 0.06–0.11), total body fat 0.06% (95% CI 0.05–0.08), visceral adipose tissue 0.08 cm^2^ (95% CI 0.04–0.12), HOMA-1B 0.06 (95% CI 0.04–0.09), and lower HDL-cholesterol levels −0.03 mmol/L (95% CI −0.05 – −0.01). In contrast, the overall depression was associated with (per s.d.) glycoprotein acetylase −0.11 mmol/L (95% CI −0.14 – −0.09), apolipoprotein B −0.04 g/L (95% CI −0.06 – −0.01), triglyceride levels −0.08 mmol/L (95% CI −0.11 – −0.06), total body fat −0.07% (95% CI −0.09 – −0.06), visceral adipose tissue −0.10 cm^2^ (95% CI −0.14 – −0.07), HOMA-1B −0.06 (95% CI −0.09 – −0.04), and HDL-cholesterol levels 0.07 mmol/L (95% CI 0.05–0.09) (Fig. 4a, 4b). We repeated the analysis of the linear regression with additional adjustment for lipid-lowering drugs (model 3) and results did not notably change (online Supplementary Tables S2, S3).

## Discussion

Using a data-driven method, we combined metabolomics and clinical symptoms data to dissect depression heterogeneity and identify independent underlying dimensions in participants diagnosed with MDD in the last 6 months from NESDA cohort (*n* = 1094). Then, we replicated our results by examining the association between the identified dimensions and 51 metabolites from the same lipidomic panel, and cardiometabolic diseases in an independent dataset of 6572 participants from the general population enrolled in the NEO study.

We used the NESDA sample including subjects with a recent MDD diagnosis to obtain a sharper picture, leveraging the higher intensity of depressive symptoms of clinical relevance, of the covariance between symptoms and metabolites commonly associated with cardiometabolic risk. We identified a major dimension reflecting overall depression explaining a large proportion (54%) of symptom-metabolite covariance, and innovatively characterized by a wide array of symptoms and reduced levels of cardiometabolic diseases. A second dimension explaining 33% of symptom-metabolite covariance emerged as characterized by higher cardiometabolic diseases and higher relative relevance for symptoms like difficulty falling asleep, sleeping too much, increase weight and appetite, low energy level and gastrointestinal problems. This second dimension partially resembles the recently postulated (Milaneschi et al., 2020) construct of IMD, defined by the clustering of inflammatory and metabolic dysregulations with behavioral energy-related symptoms. We labelled therefore the first and second dimensions ‘overall depression’ and ‘IMD’. In the replication step, we found that the IMD dimension was associated with a metabolic profile similar to the metabolic profile reported in individuals with cardiometabolic diseases such as higher triglyceride levels, visceral adipose tissue content, branched chain amino acids, glycoprotein acetylase, insulin resistance and lower HDL-cholesterol levels. In contrast, the associations between these metabolites and the overall depression dimension were in the opposite direction, indicating a lower cardiometabolic risk.

The present findings confirm the presence of partially divergent correlation structures between specific depressive symptom profiles and metabolic dysregulations. The weights estimated in NESDA certainly reduced or magnified the relevance of certain symptoms in relation to metabolic alteration. However, results obtained after weighting of the different symptoms are consistent with those obtained using unweighted depressive symptoms in previous studies. In a previous work (Alshehri et al., 2019), we investigated the association between individual depressive symptoms measured with IDS-SR30 and overall and abdominal adiposity (known proxy for adverse metabolic alteration) indexes such as total body fat, and visceral adipose tissue in NEO study. Overall, adiposity indexes were associated with a wide variety of depressive symptoms, but were more strongly associated with energy-related symptoms (i.e. hyperphagia, low energy level, and increased physical exhaustion) found to contribute relatively more strongly to the IMD-like dimension identified in the present study. Moreover, this is in line with the previous research in this field that confirmed that the presence of homeostatic shift toward increased energy (increased appetite) intake and decreased energy expenditure (sleeping too much, difficulty falling asleep (Markwald et al., 2013) and low energy level) were more strongly associated with inflammatory and metabolic biomarkers considered as risk factors for CVD. In earlier work based on NESDA data, among participants with active depression episode, increased a neuroendocrine energy homeostasis marker (leptin) (Zakrzewska, Cusin, Sainsbury, Rohner-Jeanrenaud, & Jeanrenaud, 1997) was associated (independently from BMI) with a depressive symptoms profile defined by increase in intake (increase appetite/weight) and decrease in expenditure (fatigue, low energy) (Milaneschi, Lamers, Bot, Drent, & Penninx, 2017a). Likewise, in the same population, another study confirmed the relationship between cardiometabolic diseases, such as increased abdominal adiposity, inflammation markers, and metabolic syndrome, and increased appetite during the active depressive episode (Lamers et al., 2018). In agreement with the above-mentioned well-characterized clinical cohort studies, similar results were obtained from a large population-based studies (Jokela, Virtanen, Batty, & Kivimäki, 2016) that confirmed the association between this cluster of symptoms and higher CRP. Our findings are also consistent with previous literature showing a correlation between mood-related syndrome characterized by the presence of similar atypical energy-related symptom profile and metabolic dysregulation. For example, bipolar disorder has been linked to impairment of glucose metabolism (de Melo et al., 2017), seasonal affective disorder with dysregulations of major metabolic regulator (i.e. adiponectin) (Akram et al., 2020), and sickness behavior with immuno-metabolic alterations (Capuron & Miller, 2011). Also, in a small study that combined neuroimaging and biochemical approaches, hyperphagia during depression was strongly associated with endocrine dysregulation and inflammation (Simmons et al., 2016). Interestingly, earlier (Milaneschi et al., 2017b) and recent (Badini et al., 2020) large-scale genomic studies found that the genetic overlap between BMI, CRP and leptin with depression is symptom specific; this overlap was only found in depressed patients with increased hypersomnia (Badini et al., 2020), weight and appetite (Badini et al., 2020; Milaneschi et al., 2017b). In addition, a cross-disorder systematic review identified a set of genes – coding for energy balance, metabolism, circadian rhythm, inflammation and HPA-axis activity – as potential shared genetic basis for cardiometabolic diseases, depression and bipolar disorder (Amare, Schubert, Klingler-Hoffmann, Cohen-Woods, & Baune, 2017). Another study (Adams et al., 2020) that used neuroticism as genetic specifier to stratify depression patients showed that the portion of the common genetic liability between depression and neuroticism was also shared with other psychiatric disorders; interestingly, the genetic liability not shared with neuroticism was positively correlated with metabolic phenotypes and CVD. These results confirm the existence of different dimension within the construct of depression rooted in underlying biological and genetic mechanisms. Based on evidence along this line of research, the existence of an ‘immuno-metabolic depression (IMD)’ dimension of depression was hypothesized (Milaneschi et al., 2020). This dimension is characterized by the clustering of immuno-metabolic biological alterations and behavioral symptom related to homeostasis dysregulation, which in turn can be the link between depression and CVD (Milaneschi et al., 2020).

Many plausible mechanisms can directly or indirectly lead to or result from this homeostatic shift as maintaining energy homeostasis is governed by biological, behavioral and environmental factors (Chapelot & Charlot, 2019). For example, low-grade inflammation which associated with adiposity and depression (Woelfer, Kasties, Kahlfuss, & Walter, 2019), favor − as proposed previously (Lacourt, Vichaya, Chiu, Dantzer, & Heijnen, 2018) − the fast aerobic glycolysis in the immune cells over other efficient but yet slower energy production pathways (e.g. lipid oxidation). This appropriation of the available cellular fuel done by immune cells results in low energy available to any other activities. When the body has low energy level, the circadian rhythm and sleep cycle disturb as well (i.e. feeling tired and sleeping during the day which affect sleeping time and quality during the night) (Lacourt et al., 2018). Moreover, dysregulation of neuroendocrinological signaling (e.g. leptin, and insulin which have crucial metabolic roles) may diminish their function as satiety induces hormones which lead to the development of increased appetite and decreased energy level symptoms (Chapelot & Charlot, 2019). These biological processes interact with behavioral/environmental factors that contribute in regulating of the energy homeostasis. Obesogenic environment (e.g. low physical activity demand, and availability of palatable food) could shift the energy balance toward energy accumulation which in turn can result in low grade inflammation and neuroendocrinal dysregulation (Church & Martin, 2018; Thyfault, Du, Kraus, Levine, & Booth, 2015). Putting it together, the IMD symptoms profile may reflect a prolonged homeostatic failure that closely interconnected with neuroendocrinal and metabolic dysregulation that also reported in patients with CVD (Naisberg, 1996).

Fully characterizing the IMD dimension identified in the present study, in terms of its clinical manifestation and underlying biological mechanisms is the first step in the path to a personalized approach for patients with depression (Simon & Perlis, 2010). This full characterization may help in guiding the choice of the most suitable intervention to alleviate the symptoms burden or to prevent its adverse prognosis. Moreover, understanding the clinical, and biological characteristics of this depression dimension will increase the precision of the genetic studies that aim to comprehend depression genetic architecture (Schwabe et al., 2019). Future research is needed to help us understand to what extent treating underlying metabolic dysregulation will contribute to mitigate this symptoms profile adversity. Nonetheless, we also need to know to what degree will behavioral intervention that target this symptoms profile such as exercising, dieting and sleep hygiene can improve the cardiometabolic health profile. Moreover, future genetics studies using techniques such as Mendelian randomization are needed to test the causal direction between metabolic dysregulation and specific depressive symptom profile (Kappelmann et al., 2021).

To the best of our knowledge, this study is the largest study that exploits jointly metabolomic and clinical symptom data to dissect depression dimensionality in a large, well-defined clinical (i.e. subjects with a psychiatric diagnosis) cohort (NESDA). Moreover, we replicate our findings from the clustering set in a population-based large cohort (NEO). Furthermore, while previous studies (Lamers et al., 2013; van Reedt Dortland et al., 2010) investigating the biological correlates of depression subtypes commonly examined a very limited number of biomarkers, we used an extensive lipid focused metabolomics platform (149 metabolites) and 12 cardiometabolic diseases, including four extensive adiposity measures, glucose, insulin and lipoprotein measures. While we confirmed the link between an IMD-like depression dimension and cardiometabolic risk (Milaneschi et al., 2020), a novel aspect of the present findings is that we also provided evidence of an independent dimension associated with lower cardiometabolic risk, potentially eluding to protective factors and resilience. However, some methodological issues should be considered. First, we performed the metabolite-symptom clustering and replication in two different samples. On the other hand, the samples' differences may also be considered a strength: the connection between metabolites indexing cardiometabolic risk and IMD-like depressive symptoms could be already detected in the general population, where symptom severity does not cross the clinical threshold. This may be relevant in terms of potential preventive interventions. Second, we should acknowledge the limitation of the NMR metabolite platform, which mainly is a lipidomic metabolomic platform. Accordingly, the term metabolic dysregulation should be interpreted based on the used metabolomic platform. Third, based on the cross-sectional study design, we are unable to infer the directionality of the relationship between depressive symptoms and adverse metabolic alterations.

In the present study, using a data-driven method we identified two independent depression dimensions differentially related with cardiometabolic diseases, such as higher triglycerides, higher visceral fat content, lower HDL-cholesterol levels and insulin resistance in the replication step. Our findings confirm that depression is associated with metabolic alterations that could represent the mechanism linking depression with CVD. However, these metabolic alteration are not present in all forms of depression. Depressed patients with IMD may be at higher cardiometabolic risk and may require specific additional treatment targeting underlying metabolic dysregulations.

## Supplementary material

For supplementary material accompanying this paper visit https://doi.org/10.1017/S0033291721001471. click here to view supplementary material
