# Supplementary material for: Metabolomics dissection of depression heterogeneity and related cardiometabolic risk
Source: Psychol Med. 2021 Jun 3;53(1):248–57. doi: 10.1017/S0033291721001471 (PMC9874986; doi:10.1017/S0033291721001471)
Supplement: Supplementary file 1 [file S0033291721001471sup001.docx]

**Contents of supplemntal material**

[**Appendix 1**. Peocessing the metabolmics data 2](#_Toc66202346)

[**Appendix 2.** Additional measures of body mass index (BMI), waist circumference and fasting glucose level 2](#_Toc66202347)

[**Appendix 3.** Principal component analysis (PCA) 2](#_Toc66202348)

[**Appendix 4.** Canonical correlation analysis (CCA) 3](#_Toc66202349)

[**Supplemental table 1.** Characteristics of the replication step, the Netherlands epidemiology of obesity (NEO) study 4](#_Toc66202350)

[**Supplemental figure 1**: Approaches used to parsing heterogeneity in depression. This figure is adopted from (Buch & Liston, 2021) 6](#_Toc66202351)

[**Supplemental figure 2**: Scree plot for the principle component analysis of the metabolites dataset. 6](#_Toc66202352)

[**Supplemental figure 3:** Canonical correlation between first and second canonical pairs. 7](#_Toc66202353)

[**Supplemental table 2.** The linear regression analysis of the association between the sum score of the symptoms extracted from the metabolite-symptom clustering step and the metabolites. 8](#_Toc66202354)

[**Supplemental table 3.** The linear regression analysis of the association between the sum score of the symptoms extracted from the metabolite-symptom clustering step and the cardiometabolic diseases. 20](#_Toc66202355)

[**References** 22](#_Toc66202356)

# **Appendix 1**. Peocessing the metabolmics data

Values of metabolites that could not be quantified were set as missing for all individuals. Furthermore, metabolite values with outlying concentrations (± 5 SD) were additionally set as missing. A value of 1 was added to all metabolite values, which were subsequently natural log-transformed to approximate normality. The obtained values were scaled to standard deviation units to enable comparison. This protocol for processing the metabolomic data was suggested by the manufacturer of the platform and has been consistently applied in several large-scale epidemiological studies (Bot et al., 2020; Onderwater et al., 2019). Blood samples were analyzed in two batches (April 2014 and December 2014) by ^1^H-NMR Nightingale Health Ltd, Helsinki, Finland) (Soininen, Kangas, Würtz, Suna, & Ala-Korpela, 2015). We regressed the metabolites on age and batch effect in order to remove their confounding effect.

# **Appendix 2.** Additional measures of body mass index (BMI), waist circumference and fasting glucose level

Body mass index (BMI), waist circumference and fasting glucose level were used in the analysis to examine the relationship between CCA output and cardiometabolic diseases. Height and weight were measured to calculate BMI in kg/m^2^ as an index of general adiposity. Waist circumference (cm), defined as the minimal abdominal circumference between the lower edge of the rib cage and the iliac crests, was measured by trained clinical staff according to a standardized procedure as index of abdominal adiposity. Glucose was measured from fasting plasma samples by using standard laboratory technique.

# **Appendix 3.** Principal component analysis (PCA)

PCA is an orthogonal linear transformation, that scalarly projected the data to a new coordinate system in which the maximum variation in the data projected on the first coordinate (i.e. first principal component), the second maximum variation projected on the second coordinate, and so on (Jolliffe, 2002).

# **Appendix 4.** Canonical correlation analysis (CCA)

CCA (Hotelling, 1936) is a method that, given two sets of variables X and Y (in this case, metabolites and depressive symptoms), finds a linear combination of X that is maximally correlated with a linear combination of Y (i.e., a weighted sum of each variable). The linear transformation weights were chosen such that the correlation between resulting linear combinations is maximized. These linear combinations are called canonical variates (i.e., mCV (metabolites canonical variates), sCV (symptoms canonical variates)). Together mCV and sCV are called a canonical pair and the correlation between this canonical pair is called the canonical correlation. In a specific dataset, it is possible to find multiple canonical pairs such that canonical pairs are uncorrelated to each other and equal to the number of variables in the smallest dataset. In our analysis we chose to proceed with the first two canonical pairs that provided more information about the two sets of variables. The relationship between the created canonical variables of depressive symptoms and metabolites from the same panel and cardiometabolic diseases was validated in an independent sample (see replication section).

# **Supplemental table 1.** Characteristics of the replication step, the Netherlands epidemiology of obesity (NEO) study

|  | Replication |
| --- | --- |
| Women (%) | 56.34 |
| Age (years) (mean, sd) | 55.70 (6.0) |
| High educational level (high) (%) | 46.08 |
| Tobacco smoking n% | |
| Never (%) | 38.68 |
| Former (%) | 45.25 |
| Current (%) | 16.07 |
| Alcohol consumption (g/day) median (25th ,75th percentiles) | 9.86 (2.74,21.41) |
| Use of lipid-modifying medications, (yes) (%) | 10.31 |
| BMI (kg/m^2^) (mean, sd) | 26.32(4.43) |
| Total body fat (%) (median (25th ,75th percentiles)) | 31.6 (24.8,38.2) |
| Waist circumference (cm) (mean, sd) | 92.15 (13.34) |
| Visceral adipose tissue (cm^2^) (mean, sd) | 90.04 (56.04) |
| Serum concentration of total cholesterol (mmol/L) (mean, sd) | 5.68 (1.05) |
| Serum concentration of LDL (mmol/L) (mean, sd) | 3.52 (0.96) |
| Serum concentration of HDL (mmol/L) (mean, sd) | 1.57 (0.46) |
| Serum concentration of triglyceride (mmol/L) (median (25th ,75th percentiles)) | 1.02 (0.73-1.47) |
| HBA1C (%) (mean, sd) | 5.36 (0.46) |
| Glucose (mmol/L) (mean, sd) | 5.47 (0.96) |
| HOMA-IR (median (25th ,75th percentiles)) | 1.86 (1.21,2.93) |
| HOMA 1B (median (25th ,75th percentiles)) | 86.67 (60.00,126.00) |
| Use of antidepressant Yes (%) | 6.59 |
| Total IDS-score (0-84) (median (25th ,75th percentiles)) | 8 (4,13) |

Number of individuals with data for BMI: 6572, Total body fat: 6541, Waist circumference: 6566, Visceral adipose tissue: 2537, Fasting glucose: 6554, HOMA-1B: 6541, HOMA-IR: 6545, HbA1c: 6543, Total cholesterol: 6562, HDL-cholesterol: 6561, Triglycerides: 6561, LDL-cholesterol: 6560.Normally distributed data shown as mean and standard deviation (SD), skewed distributed data shown as median (25th, 75th percentiles) and categorical data are shown as percentage. High education level: university or college education, while other education level: none, primary school or lower vocational education. IDS-SR30: Inventory of Depressive Symptomatology (self-report). BMI: body mass index. HOMA-IR: (homeostatic model assessment insulin resistance)= (glucose x insulin)/22.5, HOMA-1B: (homeostatic model assessment of beta cells) HOMA 1B=20 x insulin/glucose-3.


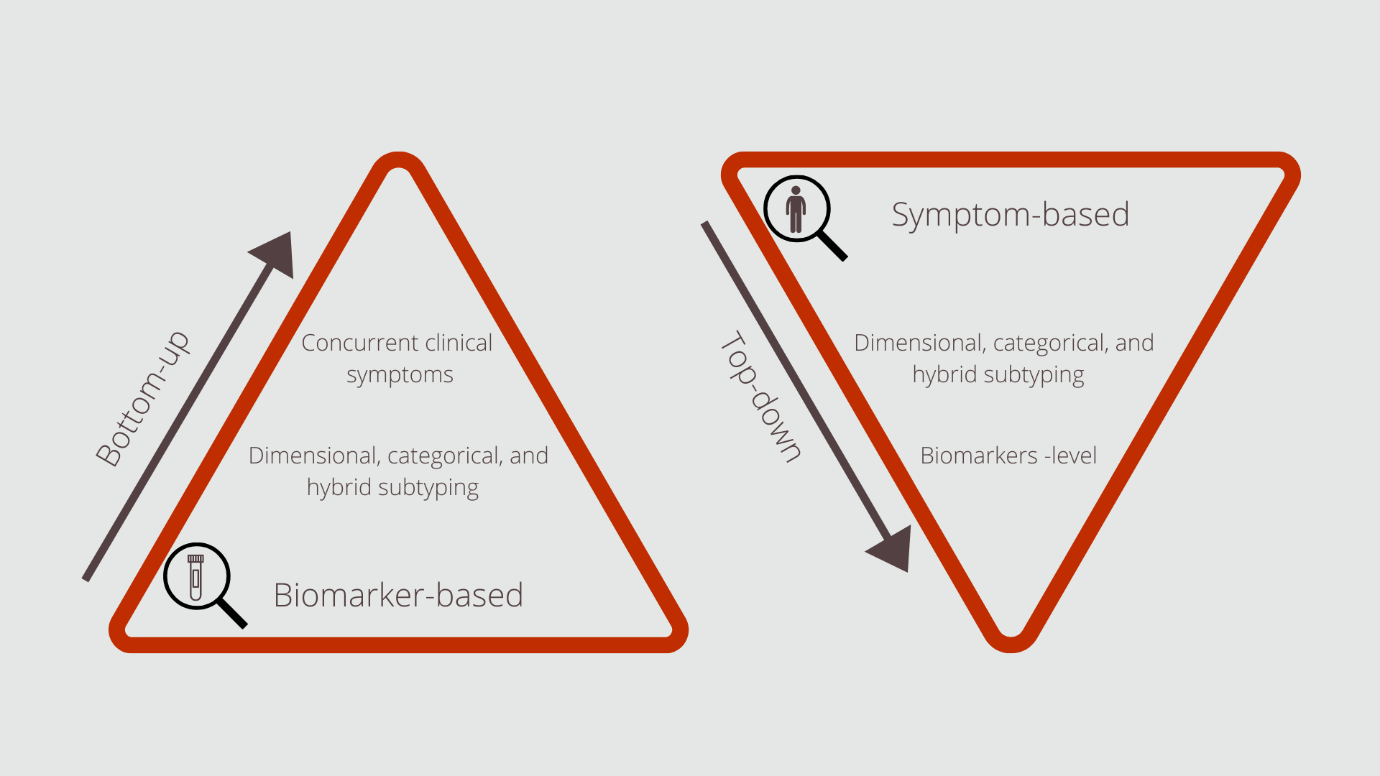


# **Supplemental figure 1**: Approaches used to parsing heterogeneity in depression. This figure is adopted from (Buch & Liston, 2021)


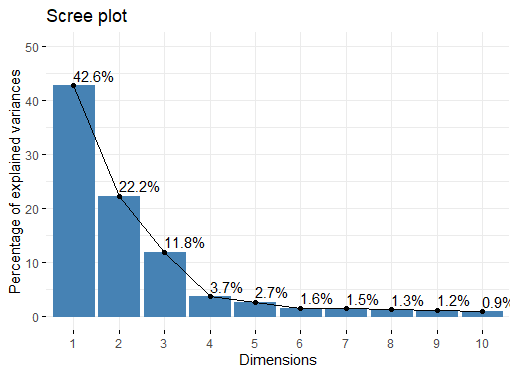


# **Supplemental figure 2**: Scree plot for the principle component analysis of the metabolites dataset.


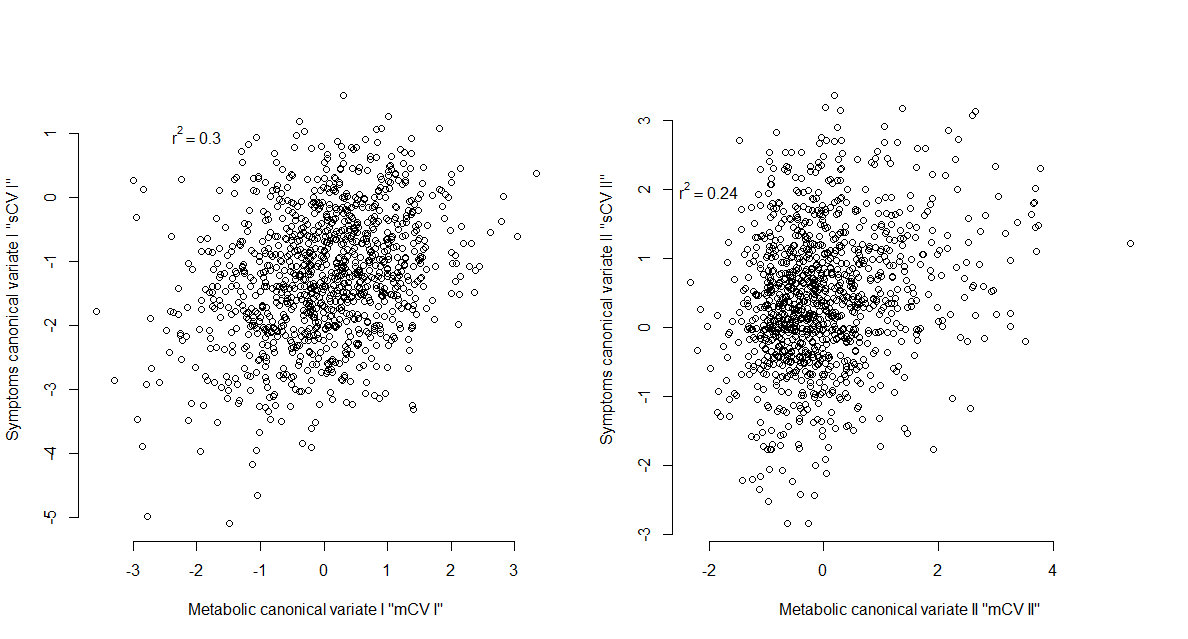


# **Supplemental figure 3:** Canonical correlation between first and second canonical pairs.

# **Supplemental table 2.** The linear regression analysis of the association between the sum score of the symptoms extracted from the metabolite-symptom clustering step and the metabolites.

|  | Crude | | | | Model 1 | | | |
| --- | --- | --- | --- | --- | --- | --- | --- | --- |
|  | Overall Depression | | IMD | | Overall Depression | | IMD | |
|  | β (95% CI) | FDR q value | β (95% CI) | FDR q value | β (95% CI) | FDR q value | β (95% CI) | FDR q value |
| XXLVLDLP | -0.08 (-0.10--0.06) | 4.78 X 10^-10^ | 0.05 (0.03-0.08) | 7.23 X 10^-05^ | -0.07 (-0.10--0.05) | 1.53 X 10^-08^ | 0.09 (0.07-0.12) | 1.96 X 10^-12^ |
| XXLVLDLL | -0.08 (-0.10--0.06) | 4.75 X 10^-10^ | 0.05 (0.03-0.08) | 7.05 X 10^-05^ | -0.07 (-0.10--0.05) | 1.53 X 10^-08^ | 0.09 (0.07-0.12) | 1.96 X 10^-12^ |
| XXLVLDLPL | -0.08 (-0.10--0.06) | 5.89 X 10^-10^ | 0.06 (0.03-0.08) | 2.08 X 10^-05^ | -0.07 (-0.10--0.05) | 1.53 X 10^-08^ | 0.09 (0.07-0.12) | 1.50 X 10^-12^ |
| XXLVLDLC | -0.07 (-0.10--0.05) | 8.09 X 10^-09^ | 0.05 (0.03-0.08) | 1.10 X 10^-04^ | -0.07 (-0.09--0.04) | 2.30 X 10^-07^ | 0.09 (0.07-0.11) | 9.40 X 10^-12^ |
| XXLVLDLCE | -0.07 (-0.09--0.04) | 1.33 X 10^-07^ | 0.04 (0.02-0.07) | 1.15 X 10^-03^ | -0.06 (-0.08--0.04) | 3.06 X 10^-06^ | 0.08 (0.06-0.10) | 1.32 X 10^-09^ |
| XXLVLDLFC | -0.08 (-0.10--0.05) | 7.96 X 10^-10^ | 0.06 (0.03-0.08) | 2.61 X 10^-05^ | -0.07 (-0.10--0.05) | 1.93 X 10^-08^ | 0.10 (0.07-0.12) | 1.24 X 10^-12^ |
| XXLVLDLTG | -0.08 (-0.10--0.06) | 3.51 X 10^-10^ | 0.05 (0.03-0.08) | 7.37 X 10^-05^ | -0.07 (-0.10--0.05) | 1.17 X 10^-08^ | 0.09 (0.07-0.12) | 1.96 X 10^-12^ |
| XLVLDLP | -0.08 (-0.11--0.06) | 2.31 X 10^-10^ | 0.05 (0.03-0.08) | 1.10 X 10^-04^ | -0.07 (-0.10--0.05) | 6.01 X 10^-09^ | 0.09 (0.07-0.12) | 1.24 X 10^-12^ |
| XLVLDLL | -0.08 (-0.11--0.06) | 1.43 X 10^-10^ | 0.05 (0.03-0.08) | 1.10 X 10^-04^ | -0.08 (-0.10--0.05) | 4.21 X 10^-09^ | 0.10 (0.07-0.12) | 1.24 X 10^-12^ |
| XLVLDLPL | -0.08 (-0.10--0.06) | 4.66 X 10^-10^ | 0.05 (0.03-0.08) | 5.26 X 10^-05^ | -0.07 (-0.10--0.05) | 1.12 X 10^-08^ | 0.09 (0.07-0.12) | 1.30 X 10^-12^ |
| XLVLDLC | -0.08 (-0.10--0.06) | 4.75 X 10^-10^ | 0.05 (0.02-0.07) | 2.78 X 10^-04^ | -0.07 (-0.10--0.05) | 1.55 X 10^-08^ | 0.09 (0.07-0.12) | 2.68 X 10^-12^ |
| XLVLDLCE | -0.08 (-0.10--0.06) | 5.90 X 10^-10^ | 0.05 (0.02-0.07) | 5.40 X 10^-04^ | -0.07 (-0.10--0.05) | 2.27 X 10^-08^ | 0.09 (0.07-0.11) | 4.39 X 10^-12^ |
| XLVLDLFC | -0.08 (-0.10--0.06) | 4.77 X 10^-10^ | 0.05 (0.03-0.08) | 8.65 X 10^-05^ | -0.07 (-0.10--0.05) | 1.33 X 10^-08^ | 0.09 (0.07-0.12) | 1.93 X 10^-12^ |
| XLVLDLTG | -0.08 (-0.11--0.06) | 6.80 X 10^-11^ | 0.05 (0.03-0.07) | 1.71 X 10^-04^ | -0.08 (-0.10--0.05) | 3.25 X 10^-09^ | 0.10 (0.07-0.12) | 1.24 X 10^-12^ |
| LVLDLP | -0.09 (-0.11--0.06) | 4.18 X 10^-11^ | 0.04 (0.02-0.07) | 8.89 X 10^-04^ | -0.08 (-0.10--0.05) | 2.11 X 10^-09^ | 0.09 (0.07-0.12) | 1.96 X 10^-12^ |
| LVLDLL | -0.09 (-0.11--0.06) | 2.05 X 10^-11^ | 0.04 (0.02-0.07) | 9.61 X 10^-04^ | -0.08 (-0.10--0.06) | 1.11 X 10^-09^ | 0.09 (0.07-0.12) | 1.82 X 10^-12^ |
| LVLDLPL | -0.09 (-0.11--0.06) | 4.18 X 10^-11^ | 0.05 (0.02-0.07) | 5.12 X 10^-04^ | -0.08 (-0.10--0.06) | 1.33 X 10^-09^ | 0.09 (0.07-0.12) | 1.96 X 10^-12^ |
| LVLDLC | -0.08 (-0.11--0.06) | 7.03 X 10^-11^ | 0.04 (0.02-0.07) | 7.50 X 10^-04^ | -0.08 (-0.10--0.05) | 2.48 X 10^-09^ | 0.09 (0.07-0.12) | 2.43 X 10^-12^ |
| LVLDLCE | -0.09 (-0.11--0.06) | 2.47 X 10^-11^ | 0.04 (0.01-0.06) | 3.73 X 10^-03^ | -0.08 (-0.10--0.05) | 1.34 X 10^-09^ | 0.09 (0.06-0.11) | 2.18 X 10^-11^ |
| LVLDLFC | -0.08 (-0.11--0.06) | 1.26 X 10^-10^ | 0.05 (0.02-0.07) | 2.13 X 10^-04^ | -0.07 (-0.10--0.05) | 6.59 X 10^-09^ | 0.09 (0.07-0.12) | 1.41 X 10^-12^ |
| LVLDLTG | -0.09 (-0.11--0.06) | 2.05 X 10^-11^ | 0.04 (0.02-0.07) | 1.29 X 10^-03^ | -0.08 (-0.10--0.06) | 1.12 X 10^-09^ | 0.09 (0.07-0.12) | 1.93 X 10^-12^ |
| MVLDLP | -0.09 (-0.11--0.06) | 2.39 X 10^-11^ | 0.03 (0.01-0.06) | 1.36 X 10^-02^ | -0.08 (-0.10--0.05) | 1.33 X 10^-09^ | 0.08 (0.06-0.11) | 5.08 X 10^-11^ |
| MVLDLL | -0.09 (-0.11--0.06) | 1.55 X 10^-11^ | 0.03 (0.01-0.06) | 1.44 X 10^-02^ | -0.08 (-0.10--0.06) | 1.11 X 10^-09^ | 0.09 (0.06-0.11) | 2.86 X 10^-11^ |
| MVLDLPL | -0.09 (-0.11--0.06) | 2.39 X 10^-11^ | 0.04 (0.01-0.06) | 7.35 X 10^-03^ | -0.08 (-0.10--0.06) | 1.33 X 10^-09^ | 0.09 (0.06-0.11) | 2.97 X 10^-11^ |
| MVLDLC | -0.08 (-0.11--0.06) | 1.02 X 10^-10^ | 0.04 (0.01-0.06) | 3.30 X 10^-03^ | -0.08 (-0.10--0.05) | 4.32 X 10^-09^ | 0.08 (0.06-0.11) | 1.20 X 10^-10^ |
| MVLDLCE | -0.08 (-0.10--0.05) | 1.34 X 10^-09^ | 0.04 (0.01-0.06) | 4.10 X 10^-03^ | -0.07 (-0.09--0.05) | 4.67 X 10^-08^ | 0.08 (0.05-0.10) | 4.13 X 10^-09^ |
| MVLDLFC | -0.09 (-0.11--0.06) | 4.72 X 10^-11^ | 0.04 (0.01-0.06) | 2.98 X 10^-03^ | -0.08 (-0.10--0.05) | 1.78 X 10^-09^ | 0.09 (0.06-0.11) | 8.61 X 10^-12^ |
| MVLDLTG | -0.09 (-0.11--0.07) | 1.28 X 10^-11^ | 0.03 (0.01-0.05) | 2.22 X 10^-02^ | -0.08 (-0.10--0.06) | 1.11 X 10^-09^ | 0.09 (0.06-0.11) | 1.07 X 10^-11^ |
| SVLDLP | -0.08 (-0.10--0.06) | 3.52 X 10^-10^ | 0.02 (0.00-0.05) | 5.85 X 10^-02^ | -0.07 (-0.10--0.05) | 1.28 X 10^-08^ | 0.07 (0.05-0.10) | 1.68 X 10^-08^ |
| SVLDLL | -0.08 (-0.10--0.06) | 5.11 X 10^-10^ | 0.02 (0.00-0.05) | 5.98 X 10^-02^ | -0.07 (-0.10--0.05) | 1.58 X 10^-08^ | 0.07 (0.05-0.10) | 2.30 X 10^-08^ |
| SVLDLPL | -0.08 (-0.10--0.05) | 1.85 X 10^-09^ | 0.03 (0.01-0.06) | 1.52 X 10^-02^ | -0.07 (-0.09--0.05) | 3.03 X 10^-08^ | 0.07 (0.05-0.10) | 2.02 X 10^-08^ |
| SVLDLC | -0.06 (-0.08--0.04) | 2.57 X 10^-06^ | 0.03 (0.00-0.05) | 5.03 X 10^-02^ | -0.05 (-0.08--0.03) | 2.62 X 10^-05^ | 0.06 (0.04-0.08) | 5.91 X 10^-06^ |
| SVLDLCE | -0.05 (-0.07--0.03) | 9.05 X 10^-05^ | 0.02 (0.00-0.04) | 1.16 X 10^-01^ | -0.05 (-0.07--0.02) | 5.31 X 10^-04^ | 0.05 (0.02-0.07) | 3.14 X 10^-04^ |
| SVLDLFC | -0.07 (-0.10--0.05) | 1.43 X 10^-08^ | 0.03 (0.01-0.06) | 1.25 X 10^-02^ | -0.07 (-0.09--0.04) | 2.09 X 10^-07^ | 0.07 (0.05-0.10) | 1.39 X 10^-08^ |
| SVLDLTG | -0.09 (-0.11--0.06) | 2.59 X 10^-11^ | 0.02 (0.00-0.05) | 8.74 X 10^-02^ | -0.08 (-0.10--0.05) | 1.34 X 10^-09^ | 0.08 (0.05-0.10) | 1.60 X 10^-09^ |
| XSVLDLP | -0.04 (-0.06--0.01) | 7.02 X 10^-03^ | 0.04 (0.02-0.07) | 8.11 X 10^-04^ | -0.04 (-0.06--0.01) | 6.04 X 10^-03^ | 0.05 (0.02-0.07) | 5.96 X 10^-04^ |
| XSVLDLL | -0.03 (-0.05--0.01) | 2.33 X 10^-02^ | 0.04 (0.02-0.07) | 1.26 X 10^-03^ | -0.03 (-0.06--0.01) | 1.85 X 10^-02^ | 0.04 (0.02-0.07) | 2.09 X 10^-03^ |
| XSVLDLPL | 0.00 (-0.03-0.02) | 7.50 X 10^-01^ | 0.05 (0.03-0.07) | 1.25 X 10^-04^ | -0.01 (-0.03-0.01) | 4.61 X 10^-01^ | 0.03 (0.00-0.05) | 3.94 X 10^-02^ |
| XSVLDLC | -0.02 (-0.04-0.01) | 1.77 X 10^-01^ | 0.03 (0.01-0.05) | 1.98 X 10^-02^ | -0.02 (-0.04-0.00) | 1.56 X 10^-01^ | 0.02 (0.00-0.05) | 8.67 X 10^-02^ |
| XSVLDLCE | -0.02 (-0.05-0.00) | 8.14 X 10^-02^ | 0.03 (0.00-0.05) | 3.24 X 10^-02^ | -0.02 (-0.05-0.00) | 8.07 X 10^-02^ | 0.02 (0.00-0.05) | 9.73 X 10^-02^ |
| XSVLDLFC | -0.01 (-0.03-0.02) | 6.72 X 10^-01^ | 0.04 (0.01-0.06) | 6.38 X 10^-03^ | -0.01 (-0.03-0.02) | 5.24 X 10^-01^ | 0.03 (0.00-0.05) | 6.42 X 10^-02^ |
| XSVLDLTG | -0.07 (-0.09--0.05) | 4.25 X 10^-08^ | 0.04 (0.01-0.06) | 4.10 X 10^-03^ | -0.07 (-0.09--0.04) | 2.39 X 10^-07^ | 0.07 (0.05-0.10) | 3.00 X 10^-08^ |
| IDLP | 0.01 (-0.01-0.04) | 3.78 X 10^-01^ | 0.05 (0.02-0.07) | 2.78 X 10^-04^ | 0.01 (-0.02-0.03) | 7.18 X 10^-01^ | 0.02 (-0.01-0.04) | 1.99 X 10^-01^ |
| IDLL | 0.02 (-0.01-0.04) | 2.30 X 10^-01^ | 0.04 (0.02-0.07) | 7.30 X 10^-04^ | 0.01 (-0.01-0.03) | 4.99 X 10^-01^ | 0.01 (-0.01-0.04) | 3.36 X 10^-01^ |
| IDLPL | 0.02 (0.00-0.05) | 6.09 X 10^-02^ | 0.04 (0.02-0.07) | 9.61 X 10^-04^ | 0.02 (-0.01-0.04) | 2.06 X 10^-01^ | 0.01 (-0.02-0.03) | 6.23 X 10^-01^ |
| IDLC | 0.02 (-0.01-0.04) | 2.00 X 10^-01^ | 0.04 (0.02-0.07) | 1.26 X 10^-03^ | 0.01 (-0.01-0.04) | 4.09 X 10^-01^ | 0.01 (-0.01-0.04) | 3.34 X 10^-01^ |
| IDLCE | 0.01 (-0.02-0.03) | 5.55 X 10^-01^ | 0.04 (0.02-0.07) | 7.00 X 10^-04^ | 0.00 (-0.02-0.03) | 7.93 X 10^-01^ | 0.02 (0.00-0.05) | 1.20 X 10^-01^ |
| IDLFC | 0.04 (0.01-0.06) | 3.12 X 10^-03^ | 0.03 (0.01-0.06) | 8.04 X 10^-03^ | 0.03 (0.01-0.05) | 2.29 X 10^-02^ | -0.01 (-0.03-0.02) | 6.87 X 10^-01^ |
| IDLTG | -0.03 (-0.06--0.01) | 1.65 X 10^-02^ | 0.07 (0.05-0.09) | 8.09 X 10^-08^ | -0.04 (-0.06--0.01) | 4.07 X 10^-03^ | 0.06 (0.03-0.08) | 2.02 X 10^-05^ |
| LLDLP | 0.02 (-0.01-0.04) | 1.83 X 10^-01^ | 0.04 (0.02-0.06) | 1.83 X 10^-03^ | 0.01 (-0.01-0.04) | 4.04 X 10^-01^ | 0.02 (-0.01-0.04) | 2.98 X 10^-01^ |
| LLDLL | 0.02 (0.00-0.04) | 1.22 X 10^-01^ | 0.04 (0.01-0.06) | 3.98 X 10^-03^ | 0.01 (-0.01-0.04) | 2.88 X 10^-01^ | 0.01 (-0.01-0.04) | 4.02 X 10^-01^ |
| LLDLPL | 0.02 (0.00-0.04) | 1.46 X 10^-01^ | 0.04 (0.02-0.07) | 1.35 X 10^-03^ | 0.01 (-0.01-0.04) | 3.42 X 10^-01^ | 0.01 (-0.01-0.04) | 3.34 X 10^-01^ |
| LLDLC | 0.02 (0.00-0.05) | 9.95 X 10^-02^ | 0.03 (0.01-0.05) | 1.95 X 10^-02^ | 0.02 (-0.01-0.04) | 2.25 X 10^-01^ | 0.01 (-0.02-0.03) | 6.17 X 10^-01^ |
| LLDLCE | 0.02 (-0.01-0.04) | 2.18 X 10^-01^ | 0.03 (0.01-0.06) | 1.52 X 10^-02^ | 0.01 (-0.01-0.04) | 3.92 X 10^-01^ | 0.01 (-0.01-0.04) | 3.83 X 10^-01^ |
| LLDLFC | 0.04 (0.01-0.06) | 3.89 X 10^-03^ | 0.03 (0.00-0.05) | 4.05 X 10^-02^ | 0.03 (0.01-0.05) | 2.24 X 10^-02^ | -0.01 (-0.03-0.02) | 6.17 X 10^-01^ |
| LLDLTG | 0.00 (-0.03-0.02) | 8.10 X 10^-01^ | 0.08 (0.06-0.11) | 2.38 X 10^-10^ | -0.01 (-0.04-0.01) | 2.82 X 10^-01^ | 0.04 (0.02-0.07) | 9.61 X 10^-04^ |
| MLDLP | 0.02 (-0.01-0.04) | 1.86 X 10^-01^ | 0.03 (0.01-0.06) | 1.58 X 10^-02^ | 0.01 (-0.01-0.04) | 3.81 X 10^-01^ | 0.01 (-0.01-0.04) | 3.80 X 10^-01^ |
| MLDLL | 0.02 (-0.01-0.04) | 1.60 X 10^-01^ | 0.03 (0.01-0.05) | 2.45 X 10^-02^ | 0.01 (-0.01-0.04) | 3.21 X 10^-01^ | 0.01 (-0.01-0.04) | 4.45 X 10^-01^ |
| MLDLPL | 0.00 (-0.02-0.03) | 8.20 X 10^-01^ | 0.04 (0.02-0.07) | 7.87 X 10^-04^ | 0.00 (-0.03-0.02) | 9.19 X 10^-01^ | 0.03 (0.00-0.05) | 3.94 X 10^-02^ |
| MLDLC | 0.02 (0.00-0.05) | 8.62 X 10^-02^ | 0.02 (0.00-0.04) | 1.35 X 10^-01^ | 0.02 (-0.01-0.04) | 1.81 X 10^-01^ | 0.00 (-0.02-0.03) | 8.36 X 10^-01^ |
| MLDLCE | 0.02 (0.00-0.05) | 9.13 X 10^-02^ | 0.02 (-0.01-0.04) | 1.78 X 10^-01^ | 0.02 (-0.01-0.04) | 1.84 X 10^-01^ | 0.00 (-0.02-0.03) | 8.71 X 10^-01^ |
| MLDLFC | 0.02 (0.00-0.05) | 6.57 X 10^-02^ | 0.03 (0.00-0.05) | 4.45 X 10^-02^ | 0.02 (-0.01-0.04) | 1.66 X 10^-01^ | 0.01 (-0.02-0.03) | 6.51 X 10^-01^ |
| MLDLTG | 0.01 (-0.02-0.03) | 5.16 X 10^-01^ | 0.09 (0.06-0.11) | 4.90 X 10^-11^ | -0.01 (-0.03-0.02) | 7.19 X 10^-01^ | 0.04 (0.01-0.06) | 4.71 X 10^-03^ |
| SLDLP | 0.02 (0.00-0.04) | 1.23 X 10^-01^ | 0.03 (0.01-0.06) | 1.47 X 10^-02^ | 0.02 (-0.01-0.04) | 2.80 X 10^-01^ | 0.01 (-0.01-0.04) | 3.64 X 10^-01^ |
| SLDLL | 0.02 (0.00-0.05) | 9.10 X 10^-02^ | 0.03 (0.01-0.05) | 2.17 X 10^-02^ | 0.02 (-0.01-0.04) | 2.11 X 10^-01^ | 0.01 (-0.01-0.04) | 4.46 X 10^-01^ |
| SLDLPL | 0.01 (-0.01-0.04) | 2.95 X 10^-01^ | 0.05 (0.02-0.07) | 2.53 X 10^-04^ | 0.01 (-0.02-0.03) | 5.67 X 10^-01^ | 0.03 (0.00-0.05) | 5.58 X 10^-02^ |
| SLDLC | 0.03 (0.00-0.05) | 3.31 X 10^-02^ | 0.02 (-0.01-0.04) | 1.84 X 10^-01^ | 0.02 (0.00-0.05) | 8.74 X 10^-02^ | 0.00 (-0.03-0.02) | 9.72 X 10^-01^ |
| SLDLCE | 0.03 (0.00-0.05) | 3.31 X 10^-02^ | 0.02 (-0.01-0.04) | 2.49 X 10^-01^ | 0.02 (0.00-0.05) | 8.54 X 10^-02^ | 0.00 (-0.03-0.02) | 8.62 X 10^-01^ |
| SLDLFC | 0.03 (0.00-0.05) | 5.39 X 10^-02^ | 0.03 (0.00-0.05) | 5.19 X 10^-02^ | 0.02 (0.00-0.04) | 1.42 X 10^-01^ | 0.01 (-0.02-0.03) | 6.49 X 10^-01^ |
| SLDLTG | -0.03 (-0.06--0.01) | 1.33 X 10^-02^ | 0.08 (0.06-0.10) | 8.23 X 10^-10^ | -0.04 (-0.06--0.01) | 4.03 X 10^-03^ | 0.07 (0.05-0.10) | 1.37 X 10^-07^ |
| XLHDLP | 0.07 (0.05-0.09) | 3.30 X 10^-08^ | 0.08 (0.06-0.10) | 9.81 X 10^-10^ | 0.05 (0.03-0.07) | 8.28 X 10^-06^ | -0.01 (-0.04-0.01) | 2.59 X 10^-01^ |
| XLHDLL | 0.07 (0.05-0.09) | 3.85 X 10^-08^ | 0.08 (0.05-0.10) | 1.48 X 10^-09^ | 0.05 (0.03-0.07) | 9.16 X 10^-06^ | -0.02 (-0.04-0.01) | 2.10 X 10^-01^ |
| XLHDLPL | 0.08 (0.05-0.10) | 1.00 X 10^-09^ | 0.07 (0.05-0.10) | 8.12 X 10^-09^ | 0.06 (0.04-0.08) | 3.74 X 10^-07^ | -0.03 (-0.05-0.00) | 3.94 X 10^-02^ |
| XLHDLC | 0.06 (0.04-0.09) | 9.77 X 10^-07^ | 0.08 (0.05-0.10) | 7.24 X 10^-09^ | 0.05 (0.02-0.07) | 1.44 X 10^-04^ | -0.01 (-0.03-0.01) | 4.30 X 10^-01^ |
| XLHDLCE | 0.06 (0.04-0.08) | 2.50 X 10^-06^ | 0.07 (0.05-0.10) | 3.81 X 10^-08^ | 0.04 (0.02-0.07) | 2.68 X 10^-04^ | -0.01 (-0.03-0.01) | 4.87 X 10^-01^ |
| XLHDLFC | 0.07 (0.04-0.09) | 1.15 X 10^-07^ | 0.08 (0.06-0.11) | 8.67 X 10^-11^ | 0.05 (0.03-0.07) | 3.25 X 10^-05^ | -0.01 (-0.03-0.01) | 4.15 X 10^-01^ |
| XLHDLTG | -0.01 (-0.04-0.01) | 3.44 X 10^-01^ | 0.09 (0.06-0.11) | 5.59 X 10^-11^ | -0.02 (-0.04-0.01) | 1.96 X 10^-01^ | 0.06 (0.03-0.08) | 1.96 X 10^-05^ |
| LHDLP | 0.08 (0.06-0.11) | 2.69 X 10^-10^ | 0.08 (0.06-0.10) | 8.60 X 10^-10^ | 0.06 (0.04-0.08) | 1.11 X 10^-07^ | -0.02 (-0.04-0.00) | 1.07 X 10^-01^ |
| LHDLL | 0.08 (0.06-0.11) | 2.43 X 10^-10^ | 0.08 (0.05-0.10) | 5.60 X 10^-09^ | 0.06 (0.04-0.08) | 1.08 X 10^-07^ | -0.02 (-0.05-0.00) | 5.61 X 10^-02^ |
| LHDLPL | 0.08 (0.06-0.10) | 4.17 X 10^-10^ | 0.08 (0.05-0.10) | 1.63 X 10^-09^ | 0.06 (0.04-0.08) | 2.28 X 10^-07^ | -0.02 (-0.04-0.00) | 9.01 X 10^-02^ |
| LHDLC | 0.08 (0.06-0.11) | 9.35 X 10^-11^ | 0.07 (0.05-0.10) | 1.06 X 10^-08^ | 0.06 (0.04-0.08) | 3.02 X 10^-08^ | -0.03 (-0.05-0.00) | 3.94 X 10^-02^ |
| LHDLCE | 0.08 (0.06-0.11) | 1.26 X 10^-10^ | 0.08 (0.05-0.10) | 7.24 X 10^-09^ | 0.06 (0.04-0.08) | 4.12 X 10^-08^ | -0.02 (-0.05-0.00) | 4.89 X 10^-02^ |
| LHDLFC | 0.08 (0.06-0.11) | 5.02 X 10^-11^ | 0.07 (0.05-0.10) | 1.34 X 10^-08^ | 0.06 (0.04-0.09) | 1.78 X 10^-08^ | -0.03 (-0.05-0.00) | 3.82 X 10^-02^ |
| LHDLTG | 0.05 (0.03-0.08) | 1.96 X 10^-05^ | 0.09 (0.07-0.12) | 1.81 X 10^-12^ | 0.04 (0.02-0.06) | 1.10 X 10^-03^ | 0.01 (-0.02-0.03) | 6.87 X 10^-01^ |
| MHDLP | 0.06 (0.03-0.08) | 1.27 X 10^-05^ | 0.10 (0.08-0.12) | 3.46 X 10^-14^ | 0.04 (0.01-0.06) | 2.67 X 10^-03^ | 0.01 (-0.01-0.03) | 4.83 X 10^-01^ |
| MHDLL | 0.05 (0.03-0.08) | 6.88 X 10^-05^ | 0.10 (0.07-0.12) | 6.45 X 10^-14^ | 0.03 (0.01-0.06) | 5.56 X 10^-03^ | 0.02 (-0.01-0.04) | 2.19 X 10^-01^ |
| MHDLPL | 0.06 (0.03-0.08) | 1.00 X 10^-05^ | 0.10 (0.08-0.13) | 9.07 X 10^-15^ | 0.04 (0.01-0.06) | 2.82 X 10^-03^ | 0.01 (-0.01-0.03) | 4.82 X 10^-01^ |
| MHDLC | 0.06 (0.04-0.09) | 8.06 X 10^-07^ | 0.08 (0.06-0.11) | 1.15 X 10^-10^ | 0.05 (0.02-0.07) | 1.44 X 10^-04^ | 0.00 (-0.02-0.03) | 9.00 X 10^-01^ |
| MHDLCE | 0.06 (0.04-0.09) | 1.06 X 10^-06^ | 0.08 (0.06-0.11) | 2.44 X 10^-10^ | 0.05 (0.02-0.07) | 1.61 X 10^-04^ | 0.00 (-0.02-0.03) | 8.96 X 10^-01^ |
| MHDLFC | 0.06 (0.04-0.09) | 4.23 X 10^-07^ | 0.09 (0.07-0.11) | 8.96 X 10^-12^ | 0.05 (0.02-0.07) | 1.26 X 10^-04^ | 0.00 (-0.02-0.02) | 9.62 X 10^-01^ |
| MHDLTG | -0.06 (-0.09--0.04) | 1.26 X 10^-06^ | 0.08 (0.05-0.10) | 4.30 X 10^-09^ | -0.06 (-0.09--0.04) | 1.50 X 10^-06^ | 0.08 (0.05-0.10) | 4.34 X 10^-09^ |
| SHDLP | 0.02 (0.00-0.05) | 9.96 X 10^-02^ | 0.06 (0.03-0.08) | 7.83 X 10^-06^ | 0.01 (-0.01-0.03) | 4.52 X 10^-01^ | 0.02 (0.00-0.05) | 1.50 X 10^-01^ |
| SHDLL | 0.03 (0.00-0.05) | 4.35 X 10^-02^ | 0.06 (0.03-0.08) | 1.25 X 10^-05^ | 0.01 (-0.01-0.04) | 2.87 X 10^-01^ | 0.02 (-0.01-0.04) | 2.49 X 10^-01^ |
| SHDLPL | 0.00 (-0.03-0.02) | 9.29 X 10^-01^ | 0.08 (0.05-0.10) | 1.43 X 10^-09^ | -0.01 (-0.04-0.01) | 4.09 X 10^-01^ | 0.04 (0.02-0.07) | 1.23 X 10^-03^ |
| SHDLC | 0.06 (0.04-0.08) | 3.78 X 10^-06^ | 0.01 (-0.01-0.04) | 2.68 X 10^-01^ | 0.05 (0.02-0.07) | 2.91 X 10^-04^ | -0.03 (-0.06--0.01) | 2.54 X 10^-02^ |
| SHDLCE | 0.06 (0.04-0.09) | 1.84 X 10^-06^ | 0.00 (-0.02-0.03) | 8.94 X 10^-01^ | 0.05 (0.03-0.07) | 9.84 X 10^-05^ | -0.04 (-0.06--0.01) | 6.27 X 10^-03^ |
| SHDLFC | 0.02 (-0.01-0.04) | 1.80 X 10^-01^ | 0.07 (0.05-0.10) | 1.86 X 10^-08^ | 0.01 (-0.02-0.03) | 6.71 X 10^-01^ | 0.03 (0.01-0.06) | 1.24 X 10^-02^ |
| SHDLTG | -0.08 (-0.10--0.05) | 7.56 X 10^-10^ | 0.03 (0.01-0.06) | 8.70 X 10^-03^ | -0.07 (-0.10--0.05) | 8.31 X 10^-09^ | 0.07 (0.05-0.10) | 1.21 X 10^-08^ |
| XXLVLDLPLp | -0.04 (-0.07--0.01) | 6.24 X 10^-03^ | 0.06 (0.04-0.09) | 1.99 X 10^-05^ | -0.04 (-0.07--0.02) | 2.22 X 10^-03^ | 0.07 (0.04-0.09) | 9.28 X 10^-06^ |
| XXLVLDLCp | -0.02 (-0.04-0.01) | 2.25 X 10^-01^ | 0.04 (0.01-0.06) | 1.17 X 10^-02^ | -0.02 (-0.04-0.01) | 3.21 X 10^-01^ | 0.06 (0.03-0.09) | 8.26 X 10^-05^ |
| XXLVLDLCEp | 0.00 (-0.03-0.03) | 9.29 X 10^-01^ | 0.02 (0.00-0.05) | 9.40 X 10^-02^ | 0.00 (-0.02-0.03) | 7.78 X 10^-01^ | 0.04 (0.01-0.06) | 1.52 X 10^-02^ |
| XXLVLDLFCp | -0.05 (-0.08--0.02) | 3.81 X 10^-04^ | 0.04 (0.01-0.07) | 6.97 X 10^-03^ | -0.05 (-0.08--0.02) | 6.16 X 10^-04^ | 0.07 (0.04-0.10) | 2.38 X 10^-06^ |
| XXLVLDLTGp | 0.03 (0.01-0.06) | 2.87 X 10^-02^ | -0.05 (-0.08--0.02) | 4.37 X 10^-04^ | 0.03 (0.00-0.06) | 3.57 X 10^-02^ | -0.07 (-0.10--0.04) | 1.85 X 10^-06^ |
| XLVLDLPLp | -0.01 (-0.04-0.01) | 4.40 X 10^-01^ | 0.05 (0.03-0.08) | 1.71 X 10^-04^ | -0.02 (-0.04-0.01) | 3.17 X 10^-01^ | 0.04 (0.01-0.07) | 4.91 X 10^-03^ |
| XLVLDLCp | -0.02 (-0.04-0.01) | 2.20 X 10^-01^ | 0.00 (-0.02-0.03) | 8.64 X 10^-01^ | -0.01 (-0.04-0.01) | 4.52 X 10^-01^ | 0.03 (0.00-0.05) | 8.70 X 10^-02^ |
| XLVLDLCEp | -0.01 (-0.03-0.02) | 6.10 X 10^-01^ | -0.02 (-0.04-0.01) | 1.89 X 10^-01^ | 0.00 (-0.03-0.03) | 9.75 X 10^-01^ | 0.01 (-0.02-0.04) | 5.12 X 10^-01^ |
| XLVLDLFCp | -0.03 (-0.06--0.01) | 2.87 X 10^-02^ | 0.03 (0.00-0.05) | 6.93 X 10^-02^ | -0.03 (-0.06-0.00) | 4.51 X 10^-02^ | 0.04 (0.01-0.07) | 5.33 X 10^-03^ |
| XLVLDLTGp | 0.02 (-0.01-0.04) | 1.85 X 10^-01^ | -0.03 (-0.06-0.00) | 4.00 X 10^-02^ | 0.02 (-0.01-0.04) | 2.89 X 10^-01^ | -0.04 (-0.07--0.01) | 5.33 X 10^-03^ |
| LVLDLPLp | 0.04 (0.02-0.07) | 9.37 X 10^-04^ | 0.06 (0.04-0.08) | 7.98 X 10^-06^ | 0.03 (0.00-0.05) | 3.28 X 10^-02^ | 0.00 (-0.02-0.03) | 8.62 X 10^-01^ |
| LVLDLCp | -0.05 (-0.07--0.02) | 4.35 X 10^-04^ | 0.02 (-0.01-0.04) | 1.90 X 10^-01^ | -0.04 (-0.07--0.02) | 1.77 X 10^-03^ | 0.05 (0.02-0.07) | 9.61 X 10^-04^ |
| LVLDLCEp | -0.02 (-0.04-0.01) | 2.41 X 10^-01^ | -0.02 (-0.04-0.01) | 1.89 X 10^-01^ | -0.01 (-0.03-0.02) | 5.28 X 10^-01^ | 0.00 (-0.02-0.03) | 8.39 X 10^-01^ |
| LVLDLFCp | -0.05 (-0.08--0.03) | 5.70 X 10^-05^ | 0.04 (0.01-0.06) | 6.95 X 10^-03^ | -0.05 (-0.08--0.03) | 1.17 X 10^-04^ | 0.06 (0.03-0.09) | 1.20 X 10^-05^ |
| LVLDLTGp | 0.04 (0.01-0.06) | 7.48 X 10^-03^ | -0.03 (-0.06--0.01) | 1.60 X 10^-02^ | 0.03 (0.01-0.06) | 1.48 X 10^-02^ | -0.05 (-0.07--0.02) | 9.61 X 10^-04^ |
| MVLDLPLp | 0.08 (0.06-0.11) | 9.12 X 10^-11^ | 0.02 (0.00-0.04) | 1.23 X 10^-01^ | 0.07 (0.04-0.09) | 5.61 X 10^-08^ | -0.05 (-0.08--0.03) | 2.16 X 10^-05^ |
| MVLDLCp | 0.01 (-0.02-0.03) | 5.28 X 10^-01^ | 0.05 (0.02-0.07) | 3.35 X 10^-04^ | 0.00 (-0.02-0.03) | 7.68 X 10^-01^ | 0.02 (-0.01-0.04) | 2.43 X 10^-01^ |
| MVLDLCEp | 0.01 (-0.02-0.03) | 6.02 X 10^-01^ | 0.03 (0.01-0.06) | 1.53 X 10^-02^ | 0.00 (-0.02-0.03) | 7.71 X 10^-01^ | 0.00 (-0.02-0.03) | 9.37 X 10^-01^ |
| MVLDLFCp | -0.05 (-0.08--0.03) | 2.28 X 10^-05^ | 0.06 (0.04-0.09) | 3.23 X 10^-06^ | -0.06 (-0.08--0.03) | 1.52 X 10^-05^ | 0.08 (0.05-0.10) | 6.06 X 10^-09^ |
| MVLDLTGp | -0.03 (-0.06--0.01) | 9.27 X 10^-03^ | -0.05 (-0.08--0.03) | 1.10 X 10^-04^ | -0.03 (-0.05-0.00) | 5.28 X 10^-02^ | 0.00 (-0.02-0.02) | 9.72 X 10^-01^ |
| SVLDLPLp | 0.07 (0.04-0.09) | 8.64 X 10^-08^ | 0.01 (-0.02-0.03) | 5.23 X 10^-01^ | 0.06 (0.03-0.08) | 5.21 X 10^-06^ | -0.05 (-0.08--0.03) | 3.24 X 10^-05^ |
| SVLDLCp | 0.03 (0.00-0.05) | 5.85 X 10^-02^ | 0.01 (-0.01-0.03) | 4.88 X 10^-01^ | 0.02 (0.00-0.05) | 1.27 X 10^-01^ | -0.01 (-0.04-0.01) | 3.35 X 10^-01^ |
| SVLDLCEp | 0.01 (-0.02-0.03) | 5.81 X 10^-01^ | 0.01 (-0.02-0.03) | 5.79 X 10^-01^ | 0.01 (-0.02-0.03) | 6.33 X 10^-01^ | 0.00 (-0.03-0.02) | 8.28 X 10^-01^ |
| SVLDLFCp | 0.08 (0.06-0.11) | 6.80 X 10^-11^ | 0.06 (0.03-0.08) | 2.11 X 10^-05^ | 0.06 (0.04-0.09) | 2.67 X 10^-07^ | -0.02 (-0.05-0.00) | 8.06 X 10^-02^ |
| SVLDLTGp | -0.07 (-0.09--0.04) | 1.33 X 10^-07^ | -0.01 (-0.04-0.01) | 3.64 X 10^-01^ | -0.06 (-0.08--0.03) | 7.55 X 10^-06^ | 0.04 (0.02-0.07) | 9.61 X 10^-04^ |
| XSVLDLPLp | 0.08 (0.06-0.11) | 1.26 X 10^-10^ | 0.02 (0.00-0.05) | 6.85 X 10^-02^ | 0.07 (0.05-0.09) | 1.55 X 10^-08^ | -0.05 (-0.07--0.02) | 2.95 X 10^-04^ |
| XSVLDLCp | 0.01 (-0.01-0.04) | 2.95 X 10^-01^ | -0.01 (-0.04-0.01) | 4.06 X 10^-01^ | 0.01 (-0.01-0.04) | 3.19 X 10^-01^ | -0.03 (-0.05-0.00) | 6.04 X 10^-02^ |
| XSVLDLCEp | -0.01 (-0.04-0.01) | 3.56 X 10^-01^ | 0.00 (-0.03-0.02) | 7.72 X 10^-01^ | -0.01 (-0.04-0.01) | 4.20 X 10^-01^ | -0.01 (-0.04-0.01) | 4.09 X 10^-01^ |
| XSVLDLFCp | 0.08 (0.05-0.10) | 1.00 X 10^-09^ | -0.02 (-0.05-0.00) | 5.85 X 10^-02^ | 0.07 (0.05-0.10) | 1.98 X 10^-08^ | -0.06 (-0.08--0.03) | 1.06 X 10^-05^ |
| XSVLDLTGp | -0.06 (-0.08--0.03) | 1.77 X 10^-05^ | 0.00 (-0.03-0.02) | 9.39 X 10^-01^ | -0.05 (-0.07--0.03) | 1.36 X 10^-04^ | 0.05 (0.02-0.07) | 6.64 X 10^-04^ |
| IDLPLp | 0.04 (0.02-0.07) | 8.34 X 10^-04^ | -0.05 (-0.08--0.03) | 1.08 X 10^-04^ | 0.04 (0.02-0.07) | 1.74 X 10^-03^ | -0.07 (-0.10--0.05) | 5.49 X 10^-08^ |
| IDLCp | 0.02 (-0.01-0.04) | 1.86 X 10^-01^ | 0.00 (-0.03-0.02) | 9.30 X 10^-01^ | 0.02 (-0.01-0.04) | 1.96 X 10^-01^ | -0.01 (-0.04-0.01) | 3.58 X 10^-01^ |
| IDLCEp | -0.03 (-0.05-0.00) | 5.50 X 10^-02^ | 0.02 (0.00-0.04) | 1.19 X 10^-01^ | -0.02 (-0.05-0.00) | 1.19 X 10^-01^ | 0.03 (0.01-0.06) | 1.52 X 10^-02^ |
| IDLFCp | 0.08 (0.05-0.10) | 7.99 X 10^-10^ | -0.02 (-0.05-0.00) | 8.36 X 10^-02^ | 0.07 (0.05-0.09) | 3.02 X 10^-08^ | -0.07 (-0.10--0.05) | 2.89 X 10^-08^ |
| IDLTGp | -0.04 (-0.07--0.02) | 8.84 X 10^-04^ | 0.01 (-0.01-0.04) | 2.70 X 10^-01^ | -0.04 (-0.07--0.02) | 1.05 X 10^-03^ | 0.04 (0.01-0.06) | 6.69 X 10^-03^ |
| LLDLPLp | -0.02 (-0.05-0.00) | 1.08 X 10^-01^ | -0.01 (-0.04-0.01) | 3.78 X 10^-01^ | -0.02 (-0.04-0.01) | 2.36 X 10^-01^ | 0.00 (-0.02-0.03) | 8.36 X 10^-01^ |
| LLDLCp | 0.03 (0.00-0.05) | 3.87 X 10^-02^ | -0.02 (-0.04-0.01) | 1.96 X 10^-01^ | 0.03 (0.00-0.05) | 3.80 X 10^-02^ | -0.02 (-0.05-0.00) | 1.53 X 10^-01^ |
| LLDLCEp | 0.00 (-0.03-0.02) | 8.70 X 10^-01^ | -0.01 (-0.03-0.02) | 6.74 X 10^-01^ | 0.00 (-0.02-0.02) | 9.97 X 10^-01^ | 0.01 (-0.02-0.03) | 7.12 X 10^-01^ |
| LLDLFCp | 0.06 (0.04-0.09) | 4.89 X 10^-07^ | -0.05 (-0.08--0.03) | 5.06 X 10^-05^ | 0.06 (0.04-0.09) | 1.55 X 10^-06^ | -0.09 (-0.11--0.06) | 2.12 X 10^-11^ |
| LLDLTGp | -0.03 (-0.05-0.00) | 5.55 X 10^-02^ | 0.05 (0.02-0.07) | 5.61 X 10^-04^ | -0.03 (-0.06--0.01) | 1.56 X 10^-02^ | 0.03 (0.01-0.06) | 1.69 X 10^-02^ |
| MLDLPLp | -0.04 (-0.06--0.02) | 2.08 X 10^-03^ | 0.01 (-0.02-0.03) | 5.61 X 10^-01^ | -0.04 (-0.06--0.01) | 7.72 X 10^-03^ | 0.03 (0.00-0.05) | 6.42 X 10^-02^ |
| MLDLCp | 0.03 (0.01-0.06) | 1.23 X 10^-02^ | -0.03 (-0.05-0.00) | 5.03 X 10^-02^ | 0.03 (0.01-0.06) | 1.33 X 10^-02^ | -0.03 (-0.05-0.00) | 3.37 X 10^-02^ |
| MLDLCEp | 0.03 (0.00-0.05) | 5.53 X 10^-02^ | -0.02 (-0.04-0.01) | 2.30 X 10^-01^ | 0.03 (0.00-0.05) | 6.40 X 10^-02^ | -0.02 (-0.05-0.00) | 1.28 X 10^-01^ |
| MLDLFCp | 0.00 (-0.03-0.02) | 9.46 X 10^-01^ | -0.03 (-0.05--0.01) | 2.48 X 10^-02^ | 0.00 (-0.02-0.03) | 8.42 X 10^-01^ | -0.02 (-0.04-0.01) | 1.85 X 10^-01^ |
| MLDLTGp | -0.01 (-0.04-0.01) | 4.23 X 10^-01^ | 0.06 (0.03-0.08) | 1.91 X 10^-05^ | -0.02 (-0.05-0.00) | 1.27 X 10^-01^ | 0.02 (0.00-0.05) | 7.60 X 10^-02^ |
| SLDLPLp | -0.03 (-0.05--0.01) | 2.17 X 10^-02^ | 0.01 (-0.01-0.04) | 2.66 X 10^-01^ | -0.03 (-0.05-0.00) | 3.91 X 10^-02^ | 0.02 (0.00-0.05) | 1.07 X 10^-01^ |
| SLDLCp | 0.03 (0.01-0.05) | 2.45 X 10^-02^ | -0.03 (-0.05--0.01) | 2.44 X 10^-02^ | 0.03 (0.00-0.05) | 3.08 X 10^-02^ | -0.04 (-0.06--0.01) | 4.96 X 10^-03^ |
| SLDLCEp | 0.02 (0.00-0.05) | 8.39 X 10^-02^ | -0.02 (-0.05-0.00) | 1.11 X 10^-01^ | 0.02 (0.00-0.05) | 1.19 X 10^-01^ | -0.03 (-0.06--0.01) | 2.14 X 10^-02^ |
| SLDLFCp | 0.00 (-0.02-0.03) | 8.40 X 10^-01^ | -0.03 (-0.05-0.00) | 4.77 X 10^-02^ | 0.01 (-0.02-0.03) | 6.23 X 10^-01^ | -0.01 (-0.04-0.01) | 4.63 X 10^-01^ |
| SLDLTGp | -0.05 (-0.07--0.02) | 2.93 X 10^-04^ | 0.06 (0.03-0.08) | 1.51 X 10^-05^ | -0.05 (-0.07--0.03) | 1.44 X 10^-04^ | 0.06 (0.04-0.09) | 5.67 X 10^-06^ |
| XLHDLPLp | 0.05 (0.03-0.08) | 5.85 X 10^-05^ | 0.01 (-0.02-0.03) | 5.00 X 10^-01^ | 0.04 (0.02-0.07) | 1.43 X 10^-03^ | -0.05 (-0.08--0.03) | 1.56 X 10^-04^ |
| XLHDLCp | -0.05 (-0.07--0.03) | 1.49 X 10^-04^ | -0.02 (-0.04-0.01) | 1.59 X 10^-01^ | -0.04 (-0.06--0.01) | 3.04 X 10^-03^ | 0.04 (0.02-0.07) | 1.14 X 10^-03^ |
| XLHDLCEp | -0.06 (-0.08--0.03) | 1.32 X 10^-05^ | -0.04 (-0.07--0.02) | 1.69 X 10^-03^ | -0.04 (-0.07--0.02) | 7.87 X 10^-04^ | 0.04 (0.01-0.06) | 3.67 X 10^-03^ |
| XLHDLFCp | 0.02 (0.00-0.05) | 9.95 X 10^-02^ | 0.08 (0.05-0.10) | 4.30 X 10^-09^ | 0.01 (-0.01-0.03) | 4.74 X 10^-01^ | 0.01 (-0.01-0.04) | 3.17 X 10^-01^ |
| XLHDLTGp | -0.07 (-0.10--0.05) | 4.07 X 10^-08^ | 0.01 (-0.01-0.04) | 4.47 X 10^-01^ | -0.06 (-0.08--0.04) | 3.55 X 10^-06^ | 0.07 (0.05-0.10) | 1.35 X 10^-08^ |
| LHDLPLp | -0.07 (-0.10--0.05) | 3.13 X 10^-08^ | -0.04 (-0.07--0.02) | 8.47 X 10^-04^ | -0.06 (-0.08--0.03) | 2.90 X 10^-06^ | 0.03 (0.01-0.05) | 1.58 X 10^-02^ |
| LHDLCp | 0.08 (0.05-0.10) | 2.25 X 10^-09^ | 0.03 (0.00-0.05) | 4.01 X 10^-02^ | 0.06 (0.04-0.09) | 5.37 X 10^-07^ | -0.05 (-0.07--0.02) | 4.72 X 10^-04^ |
| LHDLCEp | 0.07 (0.05-0.10) | 6.59 X 10^-08^ | 0.03 (0.00-0.05) | 4.24 X 10^-02^ | 0.06 (0.03-0.08) | 4.97 X 10^-06^ | -0.04 (-0.06--0.02) | 2.21 X 10^-03^ |
| LHDLFCp | 0.08 (0.06-0.11) | 2.08 X 10^-10^ | 0.02 (0.00-0.05) | 1.23 X 10^-01^ | 0.07 (0.04-0.09) | 1.25 X 10^-07^ | -0.05 (-0.08--0.03) | 6.15 X 10^-05^ |
| LHDLTGp | -0.07 (-0.09--0.04) | 2.57 X 10^-07^ | 0.01 (-0.02-0.03) | 6.37 X 10^-01^ | -0.06 (-0.08--0.03) | 2.35 X 10^-05^ | 0.05 (0.02-0.07) | 4.88 X 10^-04^ |
| MHDLPLp | -0.05 (-0.07--0.02) | 1.75 X 10^-04^ | 0.02 (0.00-0.05) | 6.85 X 10^-02^ | -0.05 (-0.07--0.02) | 1.73 X 10^-04^ | 0.03 (0.01-0.06) | 1.69 X 10^-02^ |
| MHDLCp | 0.08 (0.06-0.11) | 1.26 X 10^-10^ | -0.01 (-0.04-0.01) | 3.47 X 10^-01^ | 0.08 (0.05-0.10) | 4.32 X 10^-09^ | -0.06 (-0.08--0.03) | 2.28 X 10^-05^ |
| MHDLCEp | 0.07 (0.04-0.09) | 8.98 X 10^-08^ | -0.03 (-0.06--0.01) | 1.67 X 10^-02^ | 0.07 (0.04-0.09) | 3.42 X 10^-07^ | -0.06 (-0.08--0.03) | 3.61 X 10^-05^ |
| MHDLFCp | 0.09 (0.06-0.11) | 1.95 X 10^-11^ | 0.05 (0.03-0.07) | 1.67 X 10^-04^ | 0.07 (0.05-0.09) | 1.02 X 10^-08^ | -0.04 (-0.06--0.01) | 5.25 X 10^-03^ |
| MHDLTGp | -0.09 (-0.11--0.07) | 9.61 X 10^-12^ | 0.00 (-0.02-0.03) | 9.06 X 10^-01^ | -0.08 (-0.10--0.06) | 1.11 X 10^-09^ | 0.06 (0.03-0.08) | 9.09 X 10^-06^ |
| SHDLPLp | -0.05 (-0.07--0.02) | 4.74 X 10^-04^ | 0.05 (0.03-0.08) | 7.50 X 10^-05^ | -0.04 (-0.07--0.02) | 1.07 X 10^-03^ | 0.06 (0.03-0.08) | 3.67 X 10^-05^ |
| SHDLCp | 0.07 (0.04-0.09) | 1.24 X 10^-07^ | -0.05 (-0.07--0.02) | 2.18 X 10^-04^ | 0.06 (0.04-0.09) | 1.50 X 10^-06^ | -0.07 (-0.10--0.05) | 4.64 X 10^-08^ |
| SHDLCEp | 0.06 (0.04-0.09) | 6.10 X 10^-07^ | -0.05 (-0.08--0.03) | 1.08 X 10^-04^ | 0.06 (0.04-0.08) | 4.63 X 10^-06^ | -0.07 (-0.10--0.05) | 3.45 X 10^-08^ |
| SHDLFCp | -0.02 (-0.04-0.00) | 1.29 X 10^-01^ | 0.06 (0.04-0.09) | 2.85 X 10^-06^ | -0.02 (-0.05-0.00) | 8.73 X 10^-02^ | 0.05 (0.03-0.08) | 4.90 X 10^-05^ |
| SHDLTGp | -0.09 (-0.11--0.07) | 8.29 X 10^-12^ | 0.01 (-0.02-0.03) | 5.82 X 10^-01^ | -0.08 (-0.10--0.06) | 5.72 X 10^-10^ | 0.06 (0.04-0.09) | 6.34 X 10^-07^ |
| VLDLD | -0.10 (-0.12--0.07) | 7.36 X 10^-13^ | 0.02 (0.00-0.04) | 1.19 X 10^-01^ | -0.08 (-0.11--0.06) | 5.04 X 10^-11^ | 0.08 (0.06-0.11) | 2.86 X 10^-11^ |
| LDLD | -0.01 (-0.03-0.01) | 4.54 X 10^-01^ | 0.04 (0.02-0.06) | 2.27 X 10^-03^ | -0.01 (-0.04-0.01) | 3.97 X 10^-01^ | 0.01 (-0.02-0.03) | 7.12 X 10^-01^ |
| HDLD | 0.09 (0.06-0.11) | 4.18 X 10^-11^ | 0.07 (0.05-0.10) | 2.35 X 10^-08^ | 0.07 (0.04-0.09) | 1.17 X 10^-08^ | -0.03 (-0.05--0.01) | 1.64 X 10^-02^ |
| SerumC | 0.03 (0.01-0.05) | 2.17 X 10^-02^ | 0.06 (0.04-0.08) | 3.87 X 10^-06^ | 0.02 (0.00-0.04) | 1.30 X 10^-01^ | 0.02 (-0.01-0.04) | 2.60 X 10^-01^ |
| VLDLC | -0.07 (-0.09--0.05) | 3.18 X 10^-08^ | 0.04 (0.01-0.06) | 5.98 X 10^-03^ | -0.07 (-0.09--0.04) | 4.08 X 10^-07^ | 0.07 (0.05-0.10) | 3.75 X 10^-08^ |
| RemnantC | -0.04 (-0.06--0.02) | 2.04 X 10^-03^ | 0.04 (0.02-0.07) | 8.84 X 10^-04^ | -0.04 (-0.06--0.01) | 3.48 X 10^-03^ | 0.06 (0.03-0.08) | 3.69 X 10^-05^ |
| LDLC | 0.02 (0.00-0.05) | 6.80 X 10^-02^ | 0.02 (0.00-0.05) | 1.01 X 10^-01^ | 0.02 (0.00-0.04) | 1.61 X 10^-01^ | 0.00 (-0.02-0.03) | 9.71 X 10^-01^ |
| HDLC | 0.09 (0.07-0.12) | 2.33 X 10^-12^ | 0.07 (0.05-0.09) | 5.03 X 10^-08^ | 0.07 (0.05-0.09) | 1.34 X 10^-09^ | -0.03 (-0.05--0.01) | 1.26 X 10^-02^ |
| HDL2C | 0.09 (0.07-0.12) | 3.68 X 10^-12^ | 0.07 (0.05-0.09) | 6.01 X 10^-08^ | 0.07 (0.05-0.09) | 1.84 X 10^-09^ | -0.03 (-0.05--0.01) | 9.97 X 10^-03^ |
| HDL3C | 0.09 (0.06-0.11) | 2.10 X 10^-11^ | 0.07 (0.04-0.09) | 1.87 X 10^-07^ | 0.07 (0.04-0.09) | 3.05 X 10^-08^ | -0.02 (-0.04-0.01) | 1.71 X 10^-01^ |
| EstC | 0.04 (0.01-0.06) | 7.41 X 10^-03^ | 0.06 (0.04-0.08) | 6.35 X 10^-06^ | 0.03 (0.00-0.05) | 6.06 X 10^-02^ | 0.02 (-0.01-0.04) | 2.93 X 10^-01^ |
| FreeC | 0.02 (-0.01-0.04) | 1.79 X 10^-01^ | 0.06 (0.04-0.09) | 1.40 X 10^-06^ | 0.01 (-0.02-0.03) | 5.44 X 10^-01^ | 0.02 (-0.01-0.04) | 2.17 X 10^-01^ |
| SerumTG | -0.08 (-0.11--0.06) | 9.38 X 10^-11^ | 0.04 (0.02-0.07) | 1.06 X 10^-03^ | -0.08 (-0.10--0.05) | 1.84 X 10^-09^ | 0.09 (0.06-0.11) | 3.13 X 10^-11^ |
| VLDLTG | -0.09 (-0.11--0.06) | 2.39 X 10^-11^ | 0.03 (0.01-0.06) | 1.29 X 10^-02^ | -0.08 (-0.10--0.06) | 1.11 X 10^-09^ | 0.09 (0.06-0.11) | 2.18 X 10^-11^ |
| LDLTG | -0.01 (-0.03-0.02) | 6.28 X 10^-01^ | 0.08 (0.06-0.11) | 4.45 X 10^-10^ | -0.02 (-0.04-0.01) | 1.86 X 10^-01^ | 0.04 (0.02-0.07) | 9.32 X 10^-04^ |
| HDLTG | -0.05 (-0.08--0.03) | 4.51 X 10^-05^ | 0.09 (0.06-0.11) | 1.49 X 10^-11^ | -0.06 (-0.08--0.03) | 1.79 X 10^-05^ | 0.08 (0.05-0.10) | 2.21 X 10^-09^ |
| DAG | -0.07 (-0.10--0.04) | 6.01 X 10^-07^ | 0.02 (-0.01-0.04) | 2.36 X 10^-01^ | -0.07 (-0.09--0.04) | 2.48 X 10^-06^ | 0.04 (0.01-0.06) | 1.62 X 10^-02^ |
| DAGTG | -0.03 (-0.06-0.00) | 4.46 X 10^-02^ | -0.01 (-0.04-0.01) | 4.18 X 10^-01^ | -0.03 (-0.05-0.00) | 6.55 X 10^-02^ | -0.01 (-0.04-0.02) | 4.98 X 10^-01^ |
| TotPG | 0.01 (-0.01-0.03) | 4.65 X 10^-01^ | 0.10 (0.08-0.13) | 9.07 X 10^-15^ | 0.00 (-0.03-0.02) | 8.42 X 10^-01^ | 0.04 (0.02-0.07) | 8.41 X 10^-04^ |
| TGPG | -0.10 (-0.12--0.07) | 8.16 X 10^-13^ | 0.02 (-0.01-0.04) | 2.43 X 10^-01^ | -0.09 (-0.11--0.06) | 5.04 X 10^-11^ | 0.08 (0.06-0.10) | 3.84 X 10^-10^ |
| PC | 0.01 (-0.01-0.04) | 2.95 X 10^-01^ | 0.10 (0.08-0.13) | 9.07 X 10^-15^ | 0.00 (-0.02-0.03) | 8.47 X 10^-01^ | 0.05 (0.03-0.08) | 1.34 X 10^-04^ |
| SM | 0.02 (0.00-0.05) | 7.90 X 10^-02^ | 0.07 (0.05-0.10) | 1.85 X 10^-08^ | 0.01 (-0.02-0.03) | 6.23 X 10^-01^ | -0.01 (-0.03-0.02) | 5.74 X 10^-01^ |
| TotCho | 0.01 (-0.02-0.03) | 7.03 X 10^-01^ | 0.08 (0.05-0.10) | 6.74 X 10^-09^ | 0.00 (-0.03-0.02) | 7.31 X 10^-01^ | 0.03 (0.00-0.05) | 3.94 X 10^-02^ |
| ApoA1 | 0.07 (0.05-0.10) | 5.86 X 10^-09^ | 0.10 (0.07-0.12) | 8.76 X 10^-14^ | 0.05 (0.03-0.08) | 4.97 X 10^-06^ | 0.00 (-0.02-0.03) | 8.39 X 10^-01^ |
| ApoB | -0.04 (-0.06--0.02) | 2.59 X 10^-03^ | 0.04 (0.02-0.06) | 2.19 X 10^-03^ | -0.04 (-0.06--0.01) | 5.05 X 10^-03^ | 0.06 (0.03-0.08) | 2.26 X 10^-05^ |
| ApoBApoA1 | -0.08 (-0.10--0.05) | 2.72 X 10^-09^ | -0.01 (-0.03-0.01) | 4.81 X 10^-01^ | -0.06 (-0.09--0.04) | 3.77 X 10^-07^ | 0.05 (0.03-0.08) | 2.30 X 10^-05^ |
| TotFA | -0.04 (-0.06--0.01) | 6.72 X 10^-03^ | 0.09 (0.07-0.11) | 8.96 X 10^-12^ | -0.04 (-0.06--0.02) | 2.51 X 10^-03^ | 0.08 (0.05-0.10) | 1.68 X 10^-08^ |
| FALen | -0.04 (-0.07--0.01) | 3.73 X 10^-03^ | 0.00 (-0.02-0.03) | 8.38 X 10^-01^ | -0.04 (-0.07--0.02) | 3.48 X 10^-03^ | 0.02 (0.00-0.05) | 1.30 X 10^-01^ |
| UnSat | 0.07 (0.04-0.09) | 1.07 X 10^-07^ | -0.04 (-0.07--0.02) | 1.66 X 10^-03^ | 0.06 (0.03-0.08) | 5.21 X 10^-06^ | -0.07 (-0.10--0.05) | 3.09 X 10^-08^ |
| DHA | 0.04 (0.02-0.06) | 2.44 X 10^-03^ | 0.03 (0.01-0.06) | 1.19 X 10^-02^ | 0.02 (0.00-0.04) | 1.56 X 10^-01^ | 0.01 (-0.02-0.03) | 7.36 X 10^-01^ |
| LA | -0.02 (-0.05-0.00) | 8.83 X 10^-02^ | 0.06 (0.04-0.08) | 5.20 X 10^-06^ | -0.02 (-0.05-0.00) | 1.16 X 10^-01^ | 0.04 (0.01-0.06) | 9.05 X 10^-03^ |
| CLA | -0.02 (-0.05-0.00) | 9.44 X 10^-02^ | 0.06 (0.03-0.09) | 1.51 X 10^-05^ | -0.04 (-0.06--0.01) | 8.27 X 10^-03^ | 0.04 (0.01-0.07) | 5.33 X 10^-03^ |
| FAw3 | 0.04 (0.01-0.06) | 6.54 X 10^-03^ | 0.05 (0.02-0.07) | 2.84 X 10^-04^ | 0.02 (-0.01-0.04) | 2.56 X 10^-01^ | 0.02 (0.00-0.05) | 1.46 X 10^-01^ |
| FAw6 | -0.01 (-0.04-0.01) | 2.64 X 10^-01^ | 0.07 (0.05-0.10) | 2.35 X 10^-08^ | -0.02 (-0.04-0.01) | 1.94 X 10^-01^ | 0.04 (0.01-0.06) | 3.62 X 10^-03^ |
| PUFA | -0.01 (-0.03-0.02) | 5.82 X 10^-01^ | 0.07 (0.05-0.10) | 2.63 X 10^-08^ | -0.01 (-0.04-0.01) | 3.21 X 10^-01^ | 0.04 (0.01-0.06) | 4.91 X 10^-03^ |
| MUFA | -0.05 (-0.07--0.02) | 2.31 X 10^-04^ | 0.08 (0.05-0.10) | 3.47 X 10^-09^ | -0.05 (-0.07--0.02) | 1.96 X 10^-04^ | 0.08 (0.05-0.10) | 2.56 X 10^-09^ |
| SFA | -0.04 (-0.06--0.01) | 3.89 X 10^-03^ | 0.09 (0.07-0.12) | 1.50 X 10^-12^ | -0.04 (-0.07--0.02) | 1.52 X 10^-03^ | 0.08 (0.06-0.11) | 4.71 X 10^-10^ |
| DHAFA | 0.08 (0.05-0.10) | 3.48 X 10^-09^ | -0.02 (-0.04-0.01) | 2.23 X 10^-01^ | 0.05 (0.03-0.08) | 3.14 X 10^-05^ | -0.04 (-0.07--0.02) | 2.02 X 10^-03^ |
| LAFA | 0.02 (0.00-0.05) | 9.44 X 10^-02^ | -0.05 (-0.07--0.02) | 3.86 X 10^-04^ | 0.03 (0.01-0.06) | 1.82 X 10^-02^ | -0.06 (-0.09--0.04) | 2.63 X 10^-06^ |
| CLAFA | -0.01 (-0.03-0.02) | 5.82 X 10^-01^ | 0.03 (0.01-0.06) | 1.47 X 10^-02^ | -0.02 (-0.05-0.00) | 1.36 X 10^-01^ | 0.02 (-0.01-0.04) | 3.16 X 10^-01^ |
| FAw3FA | 0.09 (0.06-0.11) | 3.05 X 10^-11^ | -0.02 (-0.04-0.01) | 2.16 X 10^-01^ | 0.06 (0.04-0.09) | 8.03 X 10^-07^ | -0.04 (-0.07--0.02) | 2.61 X 10^-03^ |
| FAw6FA | 0.05 (0.02-0.07) | 4.44 X 10^-04^ | -0.05 (-0.07--0.02) | 2.98 X 10^-04^ | 0.05 (0.03-0.07) | 1.44 X 10^-04^ | -0.08 (-0.10--0.06) | 1.05 X 10^-09^ |
| PUFAFA | 0.06 (0.04-0.09) | 1.74 X 10^-06^ | -0.05 (-0.07--0.02) | 2.09 X 10^-04^ | 0.06 (0.03-0.08) | 4.63 X 10^-06^ | -0.08 (-0.11--0.06) | 1.33 X 10^-10^ |
| MUFAFA | -0.05 (-0.07--0.02) | 2.15 X 10^-04^ | 0.03 (0.00-0.05) | 4.18 X 10^-02^ | -0.04 (-0.07--0.02) | 6.40 X 10^-04^ | 0.06 (0.03-0.08) | 4.00 X 10^-05^ |
| SFAFA | -0.02 (-0.05-0.00) | 1.08 X 10^-01^ | 0.04 (0.01-0.06) | 7.73 X 10^-03^ | -0.02 (-0.05-0.00) | 1.10 X 10^-01^ | 0.05 (0.03-0.08) | 2.01 X 10^-04^ |
| Glc | -0.04 (-0.07--0.02) | 6.24 X 10^-04^ | 0.03 (0.01-0.05) | 2.48 X 10^-02^ | -0.06 (-0.08--0.03) | 1.34 X 10^-05^ | 0.05 (0.02-0.07) | 5.50 X 10^-04^ |
| Lac | -0.08 (-0.11--0.06) | 2.11 X 10^-10^ | 0.05 (0.03-0.08) | 1.08 X 10^-04^ | -0.08 (-0.10--0.05) | 4.25 X 10^-09^ | 0.09 (0.06-0.11) | 5.08 X 10^-11^ |
| Cit | 0.03 (0.00-0.05) | 3.31 X 10^-02^ | -0.01 (-0.03-0.02) | 6.86 X 10^-01^ | 0.01 (-0.01-0.04) | 3.89 X 10^-01^ | -0.04 (-0.07--0.02) | 1.35 X 10^-03^ |
| Ala | -0.04 (-0.06--0.02) | 1.78 X 10^-03^ | 0.08 (0.06-0.10) | 1.25 X 10^-09^ | -0.04 (-0.07--0.02) | 1.17 X 10^-03^ | 0.08 (0.06-0.11) | 3.48 X 10^-10^ |
| Gln | 0.03 (0.00-0.05) | 4.57 X 10^-02^ | -0.07 (-0.09--0.04) | 4.43 X 10^-07^ | 0.03 (0.00-0.05) | 5.46 X 10^-02^ | -0.04 (-0.07--0.02) | 1.28 X 10^-03^ |
| His | 0.01 (-0.02-0.03) | 5.85 X 10^-01^ | -0.03 (-0.05-0.00) | 4.97 X 10^-02^ | 0.02 (-0.01-0.04) | 2.44 X 10^-01^ | 0.01 (-0.01-0.04) | 3.64 X 10^-01^ |
| Ile | -0.10 (-0.13--0.08) | 3.06 X 10^-14^ | -0.02 (-0.04-0.01) | 2.04 X 10^-01^ | -0.09 (-0.11--0.06) | 1.02 X 10^-12^ | 0.08 (0.06-0.10) | 2.86 X 10^-11^ |
| Leu | -0.08 (-0.11--0.06) | 2.31 X 10^-10^ | -0.03 (-0.05--0.01) | 2.57 X 10^-02^ | -0.07 (-0.09--0.05) | 2.11 X 10^-09^ | 0.08 (0.06-0.10) | 2.12 X 10^-11^ |
| Val | -0.05 (-0.08--0.03) | 2.98 X 10^-05^ | -0.05 (-0.07--0.03) | 1.62 X 10^-04^ | -0.04 (-0.07--0.02) | 1.83 X 10^-04^ | 0.04 (0.02-0.07) | 4.16 X 10^-04^ |
| Phe | -0.07 (-0.09--0.04) | 1.15 X 10^-07^ | 0.02 (0.00-0.05) | 9.57 X 10^-02^ | -0.07 (-0.09--0.04) | 6.84 X 10^-08^ | 0.06 (0.03-0.08) | 9.20 X 10^-06^ |
| Tyr | -0.05 (-0.07--0.02) | 4.52 X 10^-04^ | 0.02 (0.00-0.05) | 1.00 X 10^-01^ | -0.05 (-0.07--0.03) | 7.94 X 10^-05^ | 0.05 (0.03-0.08) | 4.22 X 10^-05^ |
| Ace | 0.04 (0.02-0.07) | 1.01 X 10^-03^ | -0.07 (-0.09--0.04) | 1.87 X 10^-07^ | 0.04 (0.01-0.06) | 5.92 X 10^-03^ | -0.06 (-0.08--0.03) | 1.28 X 10^-05^ |
| bOHBut | 0.00 (-0.02-0.03) | 9.22 X 10^-01^ | -0.04 (-0.06--0.01) | 2.98 X 10^-03^ | -0.01 (-0.03-0.02) | 6.97 X 10^-01^ | -0.03 (-0.06--0.01) | 1.30 X 10^-02^ |
| Crea | -0.01 (-0.03-0.02) | 5.83 X 10^-01^ | -0.08 (-0.10--0.05) | 3.01 X 10^-09^ | 0.00 (-0.02-0.02) | 7.69 X 10^-01^ | 0.03 (0.01-0.06) | 2.73 X 10^-03^ |
| Alb | 0.02 (-0.01-0.04) | 1.86 X 10^-01^ | -0.02 (-0.05-0.00) | 8.70 X 10^-02^ | 0.02 (0.00-0.05) | 9.52 X 10^-02^ | 0.00 (-0.03-0.02) | 9.93 X 10^-01^ |
| Gp | -0.12 (-0.14--0.09) | 1.15 X 10^-18^ | 0.07 (0.05-0.10) | 1.89 X 10^-08^ | -0.11 (-0.14--0.09) | 5.78 X 10^-17^ | 0.08 (0.06-0.11) | 2.22 X 10^-10^ |

|  | Model 2 | | | | Model 3 | | | |
| --- | --- | --- | --- | --- | --- | --- | --- | --- |
|  | Overall Depression | | IMD | | Overall Depression | | IMD | |
|  | β (95% CI) | FDR q value | β (95% CI) | FDR q value | β (95% CI) | FDR q value | β (95% CI) | FDR q value |
| XXLVLDLP | -0.07 (-0.09--0.04) | 1.09 X 10^-06^ | 0.09 (0.06-0.11) | 3.70 X 10^-11^ | -0.05 (-0.07--0.02) | 5.79 X 10^-04^ | 0.08 (0.06-0.11) | 8.94 X 10^-10^ |
| XXLVLDLL | -0.07 (-0.09--0.04) | 1.09 X 10^-06^ | 0.09 (0.06-0.11) | 3.70 X 10^-11^ | -0.05 (-0.07--0.02) | 5.79 X 10^-04^ | 0.08 (0.06-0.11) | 8.94 X 10^-10^ |
| XXLVLDLPL | -0.07 (-0.09--0.04) | 9.36 X 10^-07^ | 0.09 (0.07-0.12) | 2.26 X 10^-11^ | -0.05 (-0.07--0.02) | 5.79 X 10^-04^ | 0.09 (0.06-0.11) | 8.94 X 10^-10^ |
| XXLVLDLC | -0.06 (-0.08--0.03) | 1.17 X 10^-05^ | 0.09 (0.06-0.11) | 1.48 X 10^-10^ | -0.04 (-0.07--0.02) | 2.16 X 10^-03^ | 0.08 (0.06-0.11) | 1.71 X 10^-09^ |
| XXLVLDLCE | -0.05 (-0.08--0.03) | 8.07 X 10^-05^ | 0.08 (0.05-0.10) | 1.64 X 10^-08^ | -0.04 (-0.06--0.01) | 5.47 X 10^-03^ | 0.07 (0.05-0.10) | 4.19 X 10^-08^ |
| XXLVLDLFC | -0.06 (-0.09--0.04) | 1.32 X 10^-06^ | 0.09 (0.07-0.12) | 2.22 X 10^-11^ | -0.05 (-0.07--0.02) | 6.72 X 10^-04^ | 0.09 (0.06-0.11) | 8.94 X 10^-10^ |
| XXLVLDLTG | -0.07 (-0.09--0.04) | 8.98 X 10^-07^ | 0.09 (0.06-0.11) | 3.58 X 10^-11^ | -0.05 (-0.07--0.02) | 5.10 X 10^-04^ | 0.08 (0.06-0.11) | 8.94 X 10^-10^ |
| XLVLDLP | -0.07 (-0.09--0.04) | 5.31 X 10^-07^ | 0.09 (0.07-0.12) | 2.22 X 10^-11^ | -0.05 (-0.08--0.03) | 4.54 X 10^-04^ | 0.08 (0.06-0.11) | 8.94 X 10^-10^ |
| XLVLDLL | -0.07 (-0.09--0.04) | 3.65 X 10^-07^ | 0.09 (0.07-0.12) | 2.22 X 10^-11^ | -0.05 (-0.08--0.03) | 3.75 X 10^-04^ | 0.08 (0.06-0.11) | 8.94 X 10^-10^ |
| XLVLDLPL | -0.07 (-0.09--0.04) | 7.59 X 10^-07^ | 0.09 (0.07-0.12) | 2.22 X 10^-11^ | -0.05 (-0.07--0.03) | 4.88 X 10^-04^ | 0.08 (0.06-0.11) | 8.94 X 10^-10^ |
| XLVLDLC | -0.06 (-0.09--0.04) | 1.32 X 10^-06^ | 0.09 (0.06-0.11) | 5.51 X 10^-11^ | -0.05 (-0.07--0.02) | 5.89 X 10^-04^ | 0.08 (0.06-0.11) | 1.52 X 10^-09^ |
| XLVLDLCE | -0.06 (-0.09--0.04) | 2.07 X 10^-06^ | 0.09 (0.06-0.11) | 8.55 X 10^-11^ | -0.05 (-0.07--0.02) | 7.00 X 10^-04^ | 0.08 (0.06-0.11) | 1.71 X 10^-09^ |
| XLVLDLFC | -0.07 (-0.09--0.04) | 9.74 X 10^-07^ | 0.09 (0.06-0.11) | 3.58 X 10^-11^ | -0.05 (-0.07--0.02) | 5.62 X 10^-04^ | 0.08 (0.06-0.11) | 9.44 X 10^-10^ |
| XLVLDLTG | -0.07 (-0.09--0.04) | 2.90 X 10^-07^ | 0.09 (0.07-0.12) | 2.22 X 10^-11^ | -0.05 (-0.08--0.03) | 3.19 X 10^-04^ | 0.08 (0.06-0.11) | 8.94 X 10^-10^ |
| LVLDLP | -0.07 (-0.09--0.04) | 2.75 X 10^-07^ | 0.09 (0.06-0.11) | 3.70 X 10^-11^ | -0.05 (-0.08--0.03) | 2.88 X 10^-04^ | 0.08 (0.06-0.10) | 1.71 X 10^-09^ |
| LVLDLL | -0.07 (-0.10--0.05) | 2.09 X 10^-07^ | 0.09 (0.06-0.11) | 3.58 X 10^-11^ | -0.06 (-0.08--0.03) | 1.96 X 10^-04^ | 0.08 (0.06-0.11) | 1.53 X 10^-09^ |
| LVLDLPL | -0.07 (-0.09--0.05) | 2.09 X 10^-07^ | 0.09 (0.06-0.11) | 3.84 X 10^-11^ | -0.05 (-0.08--0.03) | 2.14 X 10^-04^ | 0.08 (0.06-0.10) | 1.71 X 10^-09^ |
| LVLDLC | -0.07 (-0.09--0.04) | 3.19 X 10^-07^ | 0.09 (0.06-0.11) | 5.51 X 10^-11^ | -0.05 (-0.08--0.03) | 2.88 X 10^-04^ | 0.08 (0.06-0.10) | 1.71 X 10^-09^ |
| LVLDLCE | -0.07 (-0.09--0.05) | 2.29 X 10^-07^ | 0.08 (0.06-0.11) | 4.78 X 10^-10^ | -0.05 (-0.08--0.03) | 2.14 X 10^-04^ | 0.08 (0.05-0.10) | 1.04 X 10^-08^ |
| LVLDLFC | -0.07 (-0.09--0.04) | 5.67 X 10^-07^ | 0.09 (0.07-0.12) | 2.26 X 10^-11^ | -0.05 (-0.07--0.03) | 4.54 X 10^-04^ | 0.08 (0.06-0.11) | 9.44 X 10^-10^ |
| LVLDLTG | -0.07 (-0.10--0.05) | 2.09 X 10^-07^ | 0.09 (0.06-0.11) | 3.58 X 10^-11^ | -0.05 (-0.08--0.03) | 1.96 X 10^-04^ | 0.08 (0.06-0.10) | 1.71 X 10^-09^ |
| MVLDLP | -0.07 (-0.09--0.05) | 2.29 X 10^-07^ | 0.08 (0.06-0.10) | 9.78 X 10^-10^ | -0.05 (-0.08--0.03) | 2.14 X 10^-04^ | 0.07 (0.05-0.10) | 3.20 X 10^-08^ |
| MVLDLL | -0.07 (-0.09--0.05) | 2.09 X 10^-07^ | 0.08 (0.06-0.11) | 5.84 X 10^-10^ | -0.06 (-0.08--0.03) | 1.96 X 10^-04^ | 0.07 (0.05-0.10) | 1.63 X 10^-08^ |
| MVLDLPL | -0.07 (-0.09--0.05) | 2.29 X 10^-07^ | 0.08 (0.06-0.11) | 5.84 X 10^-10^ | -0.05 (-0.08--0.03) | 1.96 X 10^-04^ | 0.07 (0.05-0.10) | 1.63 X 10^-08^ |
| MVLDLC | -0.07 (-0.09--0.04) | 5.76 X 10^-07^ | 0.08 (0.05-0.10) | 2.09 X 10^-09^ | -0.05 (-0.08--0.03) | 2.59 X 10^-04^ | 0.08 (0.05-0.10) | 1.63 X 10^-08^ |
| MVLDLCE | -0.06 (-0.09--0.04) | 4.60 X 10^-06^ | 0.07 (0.05-0.10) | 6.60 X 10^-08^ | -0.05 (-0.07--0.03) | 4.88 X 10^-04^ | 0.07 (0.05-0.10) | 8.79 X 10^-08^ |
| MVLDLFC | -0.07 (-0.09--0.05) | 2.29 X 10^-07^ | 0.08 (0.06-0.11) | 1.46 X 10^-10^ | -0.05 (-0.08--0.03) | 2.33 X 10^-04^ | 0.08 (0.05-0.10) | 5.24 X 10^-09^ |
| MVLDLTG | -0.07 (-0.09--0.05) | 2.09 X 10^-07^ | 0.08 (0.06-0.11) | 2.30 X 10^-10^ | -0.05 (-0.08--0.03) | 1.96 X 10^-04^ | 0.07 (0.05-0.10) | 1.22 X 10^-08^ |
| SVLDLP | -0.06 (-0.09--0.04) | 1.78 X 10^-06^ | 0.07 (0.04-0.09) | 1.83 X 10^-07^ | -0.05 (-0.07--0.03) | 4.54 X 10^-04^ | 0.06 (0.04-0.09) | 1.28 X 10^-06^ |
| SVLDLL | -0.06 (-0.09--0.04) | 2.32 X 10^-06^ | 0.07 (0.04-0.09) | 2.18 X 10^-07^ | -0.05 (-0.07--0.03) | 4.54 X 10^-04^ | 0.06 (0.04-0.09) | 1.13 X 10^-06^ |
| SVLDLPL | -0.06 (-0.09--0.04) | 2.33 X 10^-06^ | 0.07 (0.04-0.09) | 1.50 X 10^-07^ | -0.05 (-0.07--0.03) | 4.88 X 10^-04^ | 0.07 (0.04-0.09) | 9.31 X 10^-07^ |
| SVLDLC | -0.05 (-0.07--0.02) | 6.23 X 10^-04^ | 0.06 (0.03-0.08) | 3.45 X 10^-05^ | -0.04 (-0.06--0.01) | 6.58 X 10^-03^ | 0.06 (0.03-0.08) | 1.22 X 10^-05^ |
| SVLDLCE | -0.04 (-0.06--0.01) | 5.74 X 10^-03^ | 0.04 (0.02-0.07) | 1.24 X 10^-03^ | -0.03 (-0.06--0.01) | 1.99 X 10^-02^ | 0.05 (0.03-0.07) | 1.89 X 10^-04^ |
| SVLDLFC | -0.06 (-0.08--0.03) | 1.24 X 10^-05^ | 0.07 (0.05-0.10) | 1.12 X 10^-07^ | -0.05 (-0.07--0.02) | 1.13 X 10^-03^ | 0.07 (0.04-0.09) | 4.58 X 10^-07^ |
| SVLDLTG | -0.07 (-0.09--0.04) | 2.64 X 10^-07^ | 0.07 (0.05-0.10) | 1.93 X 10^-08^ | -0.05 (-0.08--0.03) | 2.21 X 10^-04^ | 0.07 (0.04-0.09) | 5.09 X 10^-07^ |
| XSVLDLP | -0.03 (-0.06--0.01) | 2.17 X 10^-02^ | 0.04 (0.02-0.07) | 1.17 X 10^-03^ | -0.03 (-0.05-0.00) | 5.10 X 10^-02^ | 0.05 (0.03-0.08) | 1.14 X 10^-04^ |
| XSVLDLL | -0.03 (-0.05-0.00) | 5.40 X 10^-02^ | 0.04 (0.02-0.07) | 3.62 X 10^-03^ | -0.03 (-0.05-0.00) | 7.86 X 10^-02^ | 0.05 (0.02-0.07) | 2.53 X 10^-04^ |
| XSVLDLPL | -0.01 (-0.03-0.01) | 4.95 X 10^-01^ | 0.03 (0.00-0.05) | 4.38 X 10^-02^ | -0.01 (-0.04-0.01) | 4.64 X 10^-01^ | 0.04 (0.02-0.06) | 2.31 X 10^-03^ |
| XSVLDLC | -0.02 (-0.04-0.01) | 2.80 X 10^-01^ | 0.02 (0.00-0.05) | 1.17 X 10^-01^ | -0.02 (-0.04-0.01) | 2.23 X 10^-01^ | 0.03 (0.01-0.06) | 9.66 X 10^-03^ |
| XSVLDLCE | -0.02 (-0.04-0.01) | 1.89 X 10^-01^ | 0.02 (0.00-0.05) | 1.40 X 10^-01^ | -0.02 (-0.05-0.00) | 1.36 X 10^-01^ | 0.03 (0.01-0.06) | 1.35 X 10^-02^ |
| XSVLDLFC | -0.01 (-0.03-0.02) | 6.17 X 10^-01^ | 0.03 (0.00-0.05) | 7.32 X 10^-02^ | -0.01 (-0.03-0.02) | 5.68 X 10^-01^ | 0.04 (0.01-0.06) | 5.73 X 10^-03^ |
| XSVLDLTG | -0.06 (-0.08--0.03) | 1.23 X 10^-05^ | 0.07 (0.04-0.09) | 1.65 X 10^-07^ | -0.04 (-0.07--0.02) | 1.54 X 10^-03^ | 0.06 (0.04-0.09) | 1.30 X 10^-06^ |
| IDLP | 0.00 (-0.02-0.03) | 7.76 X 10^-01^ | 0.02 (-0.01-0.04) | 1.87 X 10^-01^ | 0.00 (-0.02-0.02) | 9.99 X 10^-01^ | 0.03 (0.01-0.06) | 1.04 X 10^-02^ |
| IDLL | 0.01 (-0.02-0.03) | 5.82 X 10^-01^ | 0.01 (-0.01-0.04) | 3.19 X 10^-01^ | 0.00 (-0.02-0.03) | 8.94 X 10^-01^ | 0.03 (0.01-0.05) | 1.91 X 10^-02^ |
| IDLPL | 0.01 (-0.01-0.04) | 3.00 X 10^-01^ | 0.01 (-0.02-0.03) | 5.67 X 10^-01^ | 0.01 (-0.02-0.03) | 6.14 X 10^-01^ | 0.02 (0.00-0.05) | 5.95 X 10^-02^ |
| IDLC | 0.01 (-0.01-0.03) | 4.92 X 10^-01^ | 0.01 (-0.01-0.04) | 3.15 X 10^-01^ | 0.00 (-0.02-0.03) | 8.36 X 10^-01^ | 0.03 (0.01-0.05) | 1.64 X 10^-02^ |
| IDLCE | 0.00 (-0.02-0.03) | 8.22 X 10^-01^ | 0.02 (0.00-0.05) | 1.22 X 10^-01^ | 0.00 (-0.03-0.02) | 9.03 X 10^-01^ | 0.04 (0.01-0.06) | 3.82 X 10^-03^ |
| IDLFC | 0.03 (0.00-0.05) | 5.40 X 10^-02^ | 0.00 (-0.03-0.02) | 7.74 X 10^-01^ | 0.02 (-0.01-0.04) | 2.31 X 10^-01^ | 0.01 (-0.01-0.04) | 3.26 X 10^-01^ |
| IDLTG | -0.04 (-0.06--0.01) | 6.64 X 10^-03^ | 0.06 (0.03-0.08) | 2.35 X 10^-05^ | -0.03 (-0.05-0.00) | 5.34 X 10^-02^ | 0.06 (0.03-0.08) | 1.55 X 10^-05^ |
| LLDLP | 0.01 (-0.01-0.04) | 4.76 X 10^-01^ | 0.02 (-0.01-0.04) | 2.79 X 10^-01^ | 0.01 (-0.02-0.03) | 7.23 X 10^-01^ | 0.03 (0.01-0.05) | 2.01 X 10^-02^ |
| LLDLL | 0.01 (-0.01-0.04) | 3.49 X 10^-01^ | 0.01 (-0.01-0.04) | 3.85 X 10^-01^ | 0.01 (-0.02-0.03) | 6.49 X 10^-01^ | 0.03 (0.00-0.05) | 2.86 X 10^-02^ |
| LLDLPL | 0.01 (-0.01-0.04) | 4.30 X 10^-01^ | 0.01 (-0.01-0.04) | 3.18 X 10^-01^ | 0.01 (-0.02-0.03) | 7.20 X 10^-01^ | 0.03 (0.01-0.05) | 2.14 X 10^-02^ |
| LLDLC | 0.02 (-0.01-0.04) | 2.74 X 10^-01^ | 0.01 (-0.02-0.03) | 5.89 X 10^-01^ | 0.01 (-0.02-0.03) | 5.77 X 10^-01^ | 0.02 (0.00-0.05) | 6.46 X 10^-02^ |
| LLDLCE | 0.01 (-0.01-0.04) | 4.32 X 10^-01^ | 0.01 (-0.01-0.04) | 3.85 X 10^-01^ | 0.01 (-0.02-0.03) | 7.23 X 10^-01^ | 0.03 (0.00-0.05) | 3.01 X 10^-02^ |
| LLDLFC | 0.03 (0.00-0.05) | 4.73 X 10^-02^ | -0.01 (-0.03-0.02) | 6.89 X 10^-01^ | 0.02 (-0.01-0.04) | 2.08 X 10^-01^ | 0.01 (-0.01-0.03) | 4.07 X 10^-01^ |
| LLDLTG | -0.02 (-0.04-0.01) | 2.23 X 10^-01^ | 0.05 (0.02-0.07) | 6.27 X 10^-04^ | -0.01 (-0.04-0.01) | 4.14 X 10^-01^ | 0.05 (0.03-0.08) | 1.28 X 10^-04^ |
| MLDLP | 0.01 (-0.01-0.04) | 4.41 X 10^-01^ | 0.01 (-0.01-0.04) | 3.57 X 10^-01^ | 0.01 (-0.02-0.03) | 6.51 X 10^-01^ | 0.03 (0.00-0.05) | 3.95 X 10^-02^ |
| MLDLL | 0.01 (-0.01-0.04) | 3.73 X 10^-01^ | 0.01 (-0.01-0.04) | 4.26 X 10^-01^ | 0.01 (-0.02-0.03) | 6.21 X 10^-01^ | 0.03 (0.00-0.05) | 4.80 X 10^-02^ |
| MLDLPL | 0.00 (-0.03-0.02) | 9.10 X 10^-01^ | 0.03 (0.00-0.05) | 4.25 X 10^-02^ | 0.00 (-0.03-0.02) | 8.36 X 10^-01^ | 0.04 (0.02-0.07) | 1.95 X 10^-03^ |
| MLDLC | 0.02 (-0.01-0.04) | 2.17 X 10^-01^ | 0.00 (-0.02-0.03) | 7.92 X 10^-01^ | 0.01 (-0.01-0.04) | 4.66 X 10^-01^ | 0.02 (0.00-0.04) | 1.57 X 10^-01^ |
| MLDLCE | 0.02 (-0.01-0.04) | 2.17 X 10^-01^ | 0.00 (-0.02-0.03) | 8.43 X 10^-01^ | 0.01 (-0.01-0.03) | 4.66 X 10^-01^ | 0.02 (-0.01-0.04) | 1.76 X 10^-01^ |
| MLDLFC | 0.02 (-0.01-0.04) | 2.20 X 10^-01^ | 0.01 (-0.02-0.03) | 5.82 X 10^-01^ | 0.01 (-0.01-0.04) | 4.35 X 10^-01^ | 0.02 (0.00-0.05) | 9.38 X 10^-02^ |
| MLDLTG | -0.01 (-0.03-0.01) | 4.95 X 10^-01^ | 0.04 (0.02-0.07) | 2.13 X 10^-03^ | -0.01 (-0.03-0.02) | 7.26 X 10^-01^ | 0.05 (0.02-0.07) | 5.09 X 10^-04^ |
| SLDLP | 0.01 (-0.01-0.04) | 3.47 X 10^-01^ | 0.01 (-0.01-0.04) | 3.28 X 10^-01^ | 0.01 (-0.01-0.03) | 5.42 X 10^-01^ | 0.03 (0.00-0.05) | 3.75 X 10^-02^ |
| SLDLL | 0.02 (-0.01-0.04) | 2.74 X 10^-01^ | 0.01 (-0.01-0.04) | 4.11 X 10^-01^ | 0.01 (-0.01-0.03) | 4.89 X 10^-01^ | 0.03 (0.00-0.05) | 4.82 X 10^-02^ |
| SLDLPL | 0.01 (-0.02-0.03) | 6.78 X 10^-01^ | 0.03 (0.00-0.05) | 4.86 X 10^-02^ | 0.00 (-0.02-0.03) | 8.53 X 10^-01^ | 0.04 (0.02-0.06) | 2.66 X 10^-03^ |
| SLDLC | 0.02 (0.00-0.05) | 1.21 X 10^-01^ | 0.00 (-0.02-0.03) | 9.70 X 10^-01^ | 0.01 (-0.01-0.04) | 3.18 X 10^-01^ | 0.02 (-0.01-0.04) | 2.53 X 10^-01^ |
| SLDLCE | 0.02 (0.00-0.05) | 1.16 X 10^-01^ | 0.00 (-0.03-0.02) | 8.94 X 10^-01^ | 0.01 (-0.01-0.04) | 3.17 X 10^-01^ | 0.01 (-0.01-0.04) | 3.22 X 10^-01^ |
| SLDLFC | 0.02 (-0.01-0.04) | 2.02 X 10^-01^ | 0.01 (-0.02-0.03) | 5.64 X 10^-01^ | 0.01 (-0.01-0.04) | 4.02 X 10^-01^ | 0.02 (0.00-0.05) | 1.00 X 10^-01^ |
| SLDLTG | -0.04 (-0.06--0.01) | 6.37 X 10^-03^ | 0.07 (0.05-0.10) | 1.71 X 10^-07^ | -0.03 (-0.05-0.00) | 6.25 X 10^-02^ | 0.07 (0.05-0.10) | 1.55 X 10^-07^ |
| XLHDLP | 0.04 (0.02-0.06) | 5.42 X 10^-04^ | -0.01 (-0.03-0.01) | 5.12 X 10^-01^ | 0.03 (0.01-0.05) | 1.15 X 10^-02^ | 0.00 (-0.02-0.02) | 8.17 X 10^-01^ |
| XLHDLL | 0.04 (0.02-0.06) | 5.99 X 10^-04^ | -0.01 (-0.03-0.01) | 4.18 X 10^-01^ | 0.03 (0.01-0.05) | 1.30 X 10^-02^ | 0.00 (-0.02-0.02) | 8.93 X 10^-01^ |
| XLHDLPL | 0.05 (0.02-0.07) | 7.66 X 10^-05^ | -0.02 (-0.04-0.00) | 1.27 X 10^-01^ | 0.04 (0.01-0.06) | 3.58 X 10^-03^ | -0.01 (-0.03-0.01) | 4.75 X 10^-01^ |
| XLHDLC | 0.04 (0.01-0.06) | 2.76 X 10^-03^ | -0.01 (-0.03-0.02) | 6.87 X 10^-01^ | 0.03 (0.00-0.05) | 3.51 X 10^-02^ | 0.01 (-0.01-0.03) | 5.34 X 10^-01^ |
| XLHDLCE | 0.04 (0.01-0.06) | 3.46 X 10^-03^ | 0.00 (-0.03-0.02) | 7.29 X 10^-01^ | 0.03 (0.00-0.05) | 4.10 X 10^-02^ | 0.01 (-0.01-0.03) | 4.72 X 10^-01^ |
| XLHDLFC | 0.04 (0.02-0.06) | 1.62 X 10^-03^ | 0.00 (-0.03-0.02) | 7.29 X 10^-01^ | 0.03 (0.01-0.05) | 2.21 X 10^-02^ | 0.01 (-0.02-0.03) | 6.03 X 10^-01^ |
| XLHDLTG | -0.02 (-0.04-0.01) | 2.35 X 10^-01^ | 0.06 (0.03-0.08) | 1.22 X 10^-05^ | -0.02 (-0.04-0.01) | 3.03 X 10^-01^ | 0.07 (0.04-0.09) | 4.32 X 10^-07^ |
| LHDLP | 0.05 (0.02-0.07) | 7.87 X 10^-05^ | -0.01 (-0.03-0.01) | 3.19 X 10^-01^ | 0.04 (0.01-0.06) | 3.11 X 10^-03^ | -0.01 (-0.03-0.02) | 6.67 X 10^-01^ |
| LHDLL | 0.05 (0.02-0.07) | 7.99 X 10^-05^ | -0.02 (-0.04-0.01) | 1.91 X 10^-01^ | 0.04 (0.01-0.06) | 3.41 X 10^-03^ | -0.01 (-0.03-0.01) | 4.75 X 10^-01^ |
| LHDLPL | 0.04 (0.02-0.07) | 1.61 X 10^-04^ | -0.01 (-0.04-0.01) | 2.79 X 10^-01^ | 0.03 (0.01-0.06) | 5.74 X 10^-03^ | -0.01 (-0.03-0.02) | 6.07 X 10^-01^ |
| LHDLC | 0.05 (0.03-0.07) | 2.80 X 10^-05^ | -0.02 (-0.04-0.00) | 1.46 X 10^-01^ | 0.04 (0.02-0.06) | 1.35 X 10^-03^ | -0.01 (-0.03-0.01) | 3.95 X 10^-01^ |
| LHDLCE | 0.05 (0.03-0.07) | 3.48 X 10^-05^ | -0.02 (-0.04-0.00) | 1.75 X 10^-01^ | 0.04 (0.02-0.06) | 1.59 X 10^-03^ | -0.01 (-0.03-0.01) | 4.40 X 10^-01^ |
| LHDLFC | 0.05 (0.03-0.07) | 1.81 X 10^-05^ | -0.02 (-0.04-0.00) | 1.40 X 10^-01^ | 0.04 (0.02-0.06) | 9.70 X 10^-04^ | -0.01 (-0.03-0.01) | 3.77 X 10^-01^ |
| LHDLTG | 0.03 (0.01-0.05) | 1.82 X 10^-02^ | 0.01 (-0.01-0.03) | 3.70 X 10^-01^ | 0.02 (0.00-0.04) | 8.72 X 10^-02^ | 0.02 (0.00-0.04) | 9.10 X 10^-02^ |
| MHDLP | 0.02 (0.00-0.04) | 1.12 X 10^-01^ | 0.02 (-0.01-0.04) | 1.86 X 10^-01^ | 0.02 (0.00-0.04) | 1.88 X 10^-01^ | 0.02 (0.00-0.04) | 1.66 X 10^-01^ |
| MHDLL | 0.02 (0.00-0.04) | 1.55 X 10^-01^ | 0.02 (0.00-0.05) | 7.38 X 10^-02^ | 0.02 (-0.01-0.04) | 2.60 X 10^-01^ | 0.02 (0.00-0.05) | 6.34 X 10^-02^ |
| MHDLPL | 0.02 (0.00-0.04) | 1.12 X 10^-01^ | 0.02 (-0.01-0.04) | 1.81 X 10^-01^ | 0.02 (0.00-0.04) | 1.91 X 10^-01^ | 0.02 (0.00-0.04) | 1.55 X 10^-01^ |
| MHDLC | 0.03 (0.01-0.05) | 1.82 X 10^-02^ | 0.01 (-0.01-0.03) | 5.14 X 10^-01^ | 0.02 (0.00-0.05) | 6.42 X 10^-02^ | 0.01 (-0.01-0.03) | 4.36 X 10^-01^ |
| MHDLCE | 0.03 (0.01-0.05) | 1.94 X 10^-02^ | 0.01 (-0.01-0.03) | 5.14 X 10^-01^ | 0.02 (0.00-0.05) | 6.60 X 10^-02^ | 0.01 (-0.01-0.03) | 4.45 X 10^-01^ |
| MHDLFC | 0.03 (0.01-0.05) | 1.74 X 10^-02^ | 0.01 (-0.01-0.03) | 5.44 X 10^-01^ | 0.02 (0.00-0.05) | 6.42 X 10^-02^ | 0.01 (-0.01-0.03) | 4.19 X 10^-01^ |
| MHDLTG | -0.06 (-0.09--0.04) | 7.58 X 10^-06^ | 0.08 (0.05-0.10) | 2.34 X 10^-08^ | -0.05 (-0.07--0.02) | 7.22 X 10^-04^ | 0.07 (0.04-0.09) | 2.69 X 10^-07^ |
| SHDLP | 0.00 (-0.02-0.02) | 9.90 X 10^-01^ | 0.02 (0.00-0.05) | 9.21 X 10^-02^ | 0.00 (-0.02-0.03) | 8.06 X 10^-01^ | 0.02 (-0.01-0.04) | 1.95 X 10^-01^ |
| SHDLL | 0.00 (-0.02-0.03) | 7.92 X 10^-01^ | 0.02 (0.00-0.04) | 1.57 X 10^-01^ | 0.01 (-0.02-0.03) | 7.12 X 10^-01^ | 0.02 (-0.01-0.04) | 2.72 X 10^-01^ |
| SHDLPL | -0.02 (-0.05-0.00) | 1.22 X 10^-01^ | 0.05 (0.02-0.07) | 3.64 X 10^-04^ | -0.01 (-0.04-0.01) | 4.03 X 10^-01^ | 0.04 (0.01-0.06) | 5.48 X 10^-03^ |
| SHDLC | 0.04 (0.01-0.06) | 9.12 X 10^-03^ | -0.03 (-0.05-0.00) | 5.72 X 10^-02^ | 0.03 (0.00-0.05) | 6.98 X 10^-02^ | -0.02 (-0.05-0.00) | 1.38 X 10^-01^ |
| SHDLCE | 0.04 (0.02-0.07) | 2.40 X 10^-03^ | -0.03 (-0.06--0.01) | 1.52 X 10^-02^ | 0.03 (0.01-0.06) | 3.58 X 10^-02^ | -0.03 (-0.05-0.00) | 6.16 X 10^-02^ |
| SHDLFC | -0.01 (-0.03-0.02) | 7.19 X 10^-01^ | 0.04 (0.02-0.06) | 3.06 X 10^-03^ | 0.00 (-0.02-0.03) | 9.03 X 10^-01^ | 0.03 (0.01-0.05) | 2.38 X 10^-02^ |
| SHDLTG | -0.07 (-0.09--0.04) | 6.52 X 10^-07^ | 0.07 (0.05-0.10) | 9.99 X 10^-08^ | -0.05 (-0.07--0.03) | 4.54 X 10^-04^ | 0.06 (0.04-0.09) | 4.45 X 10^-06^ |
| XXLVLDLPLp | -0.05 (-0.08--0.02) | 1.22 X 10^-03^ | 0.07 (0.04-0.10) | 7.65 X 10^-06^ | -0.04 (-0.07--0.01) | 9.66 X 10^-03^ | 0.06 (0.03-0.09) | 4.44 X 10^-05^ |
| XXLVLDLCp | -0.01 (-0.04-0.02) | 4.95 X 10^-01^ | 0.06 (0.03-0.08) | 1.86 X 10^-04^ | -0.01 (-0.03-0.02) | 7.10 X 10^-01^ | 0.06 (0.03-0.09) | 1.28 X 10^-04^ |
| XXLVLDLCEp | 0.01 (-0.02-0.04) | 6.30 X 10^-01^ | 0.04 (0.01-0.06) | 2.45 X 10^-02^ | 0.01 (-0.02-0.04) | 6.03 X 10^-01^ | 0.04 (0.01-0.07) | 6.41 X 10^-03^ |
| XXLVLDLFCp | -0.05 (-0.07--0.02) | 1.62 X 10^-03^ | 0.07 (0.04-0.10) | 4.84 X 10^-06^ | -0.04 (-0.07--0.01) | 1.19 X 10^-02^ | 0.06 (0.03-0.09) | 3.71 X 10^-05^ |
| XXLVLDLTGp | 0.03 (0.00-0.06) | 6.34 X 10^-02^ | -0.07 (-0.10--0.04) | 4.82 X 10^-06^ | 0.02 (-0.01-0.05) | 1.94 X 10^-01^ | -0.07 (-0.10--0.04) | 5.83 X 10^-06^ |
| XLVLDLPLp | -0.02 (-0.04-0.01) | 2.74 X 10^-01^ | 0.05 (0.02-0.07) | 2.48 X 10^-03^ | -0.01 (-0.04-0.01) | 4.31 X 10^-01^ | 0.05 (0.02-0.08) | 1.05 X 10^-03^ |
| XLVLDLCp | -0.01 (-0.03-0.02) | 6.13 X 10^-01^ | 0.02 (0.00-0.05) | 1.16 X 10^-01^ | -0.01 (-0.03-0.02) | 7.64 X 10^-01^ | 0.03 (0.00-0.06) | 4.79 X 10^-02^ |
| XLVLDLCEp | 0.01 (-0.02-0.03) | 7.30 X 10^-01^ | 0.01 (-0.02-0.04) | 6.00 X 10^-01^ | 0.01 (-0.02-0.03) | 7.40 X 10^-01^ | 0.02 (-0.01-0.04) | 3.04 X 10^-01^ |
| XLVLDLFCp | -0.03 (-0.06-0.00) | 5.47 X 10^-02^ | 0.04 (0.01-0.07) | 6.56 X 10^-03^ | -0.02 (-0.05-0.00) | 1.49 X 10^-01^ | 0.04 (0.02-0.07) | 4.69 X 10^-03^ |
| XLVLDLTGp | 0.01 (-0.01-0.04) | 3.51 X 10^-01^ | -0.04 (-0.07--0.01) | 6.70 X 10^-03^ | 0.01 (-0.02-0.04) | 4.91 X 10^-01^ | -0.05 (-0.07--0.02) | 1.68 X 10^-03^ |
| LVLDLPLp | 0.02 (0.00-0.05) | 8.41 X 10^-02^ | 0.01 (-0.02-0.03) | 6.87 X 10^-01^ | 0.02 (0.00-0.04) | 1.88 X 10^-01^ | 0.01 (-0.01-0.04) | 3.04 X 10^-01^ |
| LVLDLCp | -0.03 (-0.06--0.01) | 1.50 X 10^-02^ | 0.04 (0.01-0.07) | 3.92 X 10^-03^ | -0.03 (-0.05-0.00) | 6.12 X 10^-02^ | 0.04 (0.02-0.07) | 2.00 X 10^-03^ |
| LVLDLCEp | 0.00 (-0.03-0.02) | 9.57 X 10^-01^ | 0.00 (-0.03-0.02) | 8.64 X 10^-01^ | 0.00 (-0.03-0.02) | 9.27 X 10^-01^ | 0.01 (-0.02-0.03) | 6.27 X 10^-01^ |
| LVLDLFCp | -0.05 (-0.07--0.02) | 7.91 X 10^-04^ | 0.06 (0.03-0.08) | 3.47 X 10^-05^ | -0.04 (-0.06--0.01) | 1.02 X 10^-02^ | 0.05 (0.03-0.08) | 1.03 X 10^-04^ |
| LVLDLTGp | 0.03 (0.00-0.05) | 5.04 X 10^-02^ | -0.04 (-0.07--0.02) | 2.44 X 10^-03^ | 0.02 (0.00-0.05) | 1.32 X 10^-01^ | -0.05 (-0.07--0.02) | 6.68 X 10^-04^ |
| MVLDLPLp | 0.06 (0.03-0.08) | 9.66 X 10^-06^ | -0.05 (-0.07--0.02) | 1.65 X 10^-04^ | 0.04 (0.02-0.07) | 1.10 X 10^-03^ | -0.04 (-0.06--0.02) | 2.36 X 10^-03^ |
| MVLDLCp | 0.01 (-0.02-0.03) | 6.69 X 10^-01^ | 0.02 (-0.01-0.04) | 2.00 X 10^-01^ | 0.00 (-0.02-0.03) | 9.09 X 10^-01^ | 0.03 (0.01-0.06) | 1.64 X 10^-02^ |
| MVLDLCEp | 0.01 (-0.02-0.03) | 6.78 X 10^-01^ | 0.00 (-0.02-0.03) | 8.94 X 10^-01^ | 0.00 (-0.02-0.02) | 9.47 X 10^-01^ | 0.02 (-0.01-0.04) | 2.53 X 10^-01^ |
| MVLDLFCp | -0.05 (-0.08--0.03) | 9.79 X 10^-05^ | 0.08 (0.05-0.10) | 5.39 X 10^-09^ | -0.04 (-0.06--0.01) | 5.22 X 10^-03^ | 0.07 (0.05-0.10) | 8.11 X 10^-08^ |
| MVLDLTGp | -0.02 (-0.05-0.00) | 8.00 X 10^-02^ | 0.00 (-0.03-0.02) | 8.08 X 10^-01^ | -0.02 (-0.04-0.01) | 2.46 X 10^-01^ | -0.02 (-0.04-0.01) | 1.74 X 10^-01^ |
| SVLDLPLp | 0.04 (0.02-0.07) | 7.44 X 10^-04^ | -0.05 (-0.07--0.02) | 3.35 X 10^-04^ | 0.04 (0.01-0.06) | 7.94 X 10^-03^ | -0.05 (-0.07--0.02) | 3.44 X 10^-04^ |
| SVLDLCp | 0.02 (0.00-0.05) | 1.19 X 10^-01^ | -0.01 (-0.04-0.01) | 3.23 X 10^-01^ | 0.01 (-0.01-0.04) | 4.10 X 10^-01^ | 0.00 (-0.02-0.02) | 9.33 X 10^-01^ |
| SVLDLCEp | 0.01 (-0.02-0.03) | 4.95 X 10^-01^ | -0.01 (-0.03-0.02) | 7.29 X 10^-01^ | 0.00 (-0.02-0.03) | 8.99 X 10^-01^ | 0.01 (-0.02-0.03) | 5.07 X 10^-01^ |
| SVLDLFCp | 0.05 (0.03-0.07) | 5.74 X 10^-05^ | -0.02 (-0.04-0.01) | 2.55 X 10^-01^ | 0.04 (0.02-0.07) | 9.65 X 10^-04^ | -0.01 (-0.03-0.01) | 4.46 X 10^-01^ |
| SVLDLTGp | -0.05 (-0.08--0.03) | 6.75 X 10^-05^ | 0.04 (0.02-0.07) | 2.23 X 10^-03^ | -0.04 (-0.06--0.02) | 3.16 X 10^-03^ | 0.02 (0.00-0.05) | 5.21 X 10^-02^ |
| XSVLDLPLp | 0.06 (0.03-0.08) | 1.04 X 10^-05^ | -0.04 (-0.07--0.02) | 1.16 X 10^-03^ | 0.04 (0.02-0.06) | 1.61 X 10^-03^ | -0.03 (-0.05--0.01) | 2.45 X 10^-02^ |
| XSVLDLCp | 0.02 (-0.01-0.04) | 2.80 X 10^-01^ | -0.03 (-0.05-0.00) | 6.35 X 10^-02^ | 0.00 (-0.02-0.03) | 8.10 X 10^-01^ | -0.01 (-0.04-0.01) | 4.06 X 10^-01^ |
| XSVLDLCEp | -0.01 (-0.03-0.02) | 6.59 X 10^-01^ | -0.01 (-0.04-0.01) | 3.84 X 10^-01^ | -0.02 (-0.04-0.01) | 2.87 X 10^-01^ | 0.00 (-0.02-0.02) | 9.96 X 10^-01^ |
| XSVLDLFCp | 0.06 (0.04-0.09) | 2.50 X 10^-06^ | -0.06 (-0.08--0.03) | 4.40 X 10^-05^ | 0.05 (0.02-0.07) | 6.05 X 10^-04^ | -0.04 (-0.06--0.02) | 2.66 X 10^-03^ |
| XSVLDLTGp | -0.05 (-0.07--0.02) | 6.21 X 10^-04^ | 0.04 (0.02-0.07) | 1.09 X 10^-03^ | -0.03 (-0.05--0.01) | 2.65 X 10^-02^ | 0.03 (0.00-0.05) | 4.64 X 10^-02^ |
| IDLPLp | 0.03 (0.01-0.06) | 1.17 X 10^-02^ | -0.07 (-0.10--0.05) | 1.44 X 10^-07^ | 0.03 (0.00-0.05) | 7.63 X 10^-02^ | -0.07 (-0.10--0.05) | 4.40 X 10^-08^ |
| IDLCp | 0.02 (-0.01-0.04) | 2.04 X 10^-01^ | -0.01 (-0.04-0.01) | 3.42 X 10^-01^ | 0.01 (-0.02-0.03) | 7.08 X 10^-01^ | 0.00 (-0.02-0.03) | 7.47 X 10^-01^ |
| IDLCEp | -0.02 (-0.04-0.01) | 2.74 X 10^-01^ | 0.03 (0.01-0.06) | 2.77 X 10^-02^ | -0.02 (-0.04-0.01) | 2.03 X 10^-01^ | 0.04 (0.02-0.07) | 1.55 X 10^-03^ |
| IDLFCp | 0.06 (0.04-0.09) | 2.97 X 10^-06^ | -0.07 (-0.09--0.04) | 1.91 X 10^-07^ | 0.04 (0.02-0.07) | 1.15 X 10^-03^ | -0.05 (-0.08--0.03) | 5.16 X 10^-05^ |
| IDLTGp | -0.04 (-0.07--0.02) | 2.47 X 10^-03^ | 0.04 (0.01-0.06) | 8.07 X 10^-03^ | -0.03 (-0.05-0.00) | 4.50 X 10^-02^ | 0.02 (-0.01-0.04) | 1.76 X 10^-01^ |
| LLDLPLp | -0.02 (-0.04-0.01) | 2.74 X 10^-01^ | 0.00 (-0.02-0.03) | 8.74 X 10^-01^ | -0.01 (-0.03-0.02) | 5.87 X 10^-01^ | -0.01 (-0.04-0.01) | 2.94 X 10^-01^ |
| LLDLCp | 0.03 (0.00-0.05) | 4.37 X 10^-02^ | -0.02 (-0.05-0.00) | 1.49 X 10^-01^ | 0.02 (-0.01-0.04) | 2.46 X 10^-01^ | 0.00 (-0.03-0.02) | 8.23 X 10^-01^ |
| LLDLCEp | 0.00 (-0.02-0.03) | 8.22 X 10^-01^ | 0.00 (-0.02-0.03) | 8.11 X 10^-01^ | 0.00 (-0.03-0.02) | 8.94 X 10^-01^ | 0.02 (-0.01-0.04) | 1.74 X 10^-01^ |
| LLDLFCp | 0.05 (0.03-0.08) | 5.81 X 10^-05^ | -0.08 (-0.11--0.06) | 2.67 X 10^-10^ | 0.04 (0.01-0.06) | 7.72 X 10^-03^ | -0.08 (-0.10--0.05) | 1.22 X 10^-08^ |
| LLDLTGp | -0.03 (-0.06--0.01) | 1.44 X 10^-02^ | 0.03 (0.01-0.06) | 1.36 X 10^-02^ | -0.02 (-0.05-0.00) | 1.50 X 10^-01^ | 0.02 (-0.01-0.04) | 1.63 X 10^-01^ |
| MLDLPLp | -0.03 (-0.06--0.01) | 1.19 X 10^-02^ | 0.03 (0.00-0.05) | 7.32 X 10^-02^ | -0.02 (-0.05-0.00) | 7.75 X 10^-02^ | 0.01 (-0.02-0.03) | 5.24 X 10^-01^ |
| MLDLCp | 0.03 (0.01-0.06) | 1.58 X 10^-02^ | -0.03 (-0.06-0.00) | 3.28 X 10^-02^ | 0.02 (0.00-0.05) | 1.10 X 10^-01^ | -0.01 (-0.04-0.01) | 2.94 X 10^-01^ |
| MLDLCEp | 0.03 (0.00-0.05) | 7.50 X 10^-02^ | -0.02 (-0.05-0.00) | 1.22 X 10^-01^ | 0.02 (-0.01-0.04) | 2.60 X 10^-01^ | -0.01 (-0.03-0.02) | 6.21 X 10^-01^ |
| MLDLFCp | 0.00 (-0.02-0.03) | 9.27 X 10^-01^ | -0.02 (-0.04-0.01) | 2.40 X 10^-01^ | 0.01 (-0.02-0.03) | 7.03 X 10^-01^ | -0.03 (-0.06--0.01) | 1.72 X 10^-02^ |
| MLDLTGp | -0.02 (-0.05-0.00) | 8.00 X 10^-02^ | 0.03 (0.00-0.05) | 5.58 X 10^-02^ | -0.01 (-0.04-0.01) | 3.42 X 10^-01^ | 0.01 (-0.01-0.04) | 3.09 X 10^-01^ |
| SLDLPLp | -0.03 (-0.05-0.00) | 3.40 X 10^-02^ | 0.02 (0.00-0.05) | 9.90 X 10^-02^ | -0.02 (-0.04-0.00) | 1.66 X 10^-01^ | 0.01 (-0.02-0.03) | 6.21 X 10^-01^ |
| SLDLCp | 0.03 (0.00-0.05) | 3.39 X 10^-02^ | -0.04 (-0.07--0.01) | 4.76 X 10^-03^ | 0.02 (-0.01-0.04) | 2.23 X 10^-01^ | -0.02 (-0.05-0.00) | 9.10 X 10^-02^ |
| SLDLCEp | 0.02 (0.00-0.05) | 1.29 X 10^-01^ | -0.03 (-0.06--0.01) | 2.01 X 10^-02^ | 0.01 (-0.01-0.03) | 4.89 X 10^-01^ | -0.02 (-0.04-0.01) | 2.39 X 10^-01^ |
| SLDLFCp | 0.01 (-0.02-0.03) | 7.09 X 10^-01^ | -0.01 (-0.03-0.02) | 5.89 X 10^-01^ | 0.01 (-0.01-0.03) | 5.13 X 10^-01^ | -0.02 (-0.05-0.00) | 1.18 X 10^-01^ |
| SLDLTGp | -0.05 (-0.07--0.02) | 2.42 X 10^-04^ | 0.06 (0.04-0.09) | 6.31 X 10^-06^ | -0.03 (-0.06--0.01) | 1.58 X 10^-02^ | 0.05 (0.02-0.07) | 5.94 X 10^-04^ |
| XLHDLPLp | 0.03 (0.01-0.06) | 1.82 X 10^-02^ | -0.05 (-0.07--0.02) | 4.78 X 10^-04^ | 0.03 (0.00-0.05) | 7.28 X 10^-02^ | -0.05 (-0.07--0.02) | 6.83 X 10^-04^ |
| XLHDLCp | -0.03 (-0.05-0.00) | 5.40 X 10^-02^ | 0.04 (0.01-0.06) | 5.11 X 10^-03^ | -0.02 (-0.05-0.00) | 1.10 X 10^-01^ | 0.04 (0.02-0.07) | 1.70 X 10^-03^ |
| XLHDLCEp | -0.03 (-0.05-0.00) | 3.24 X 10^-02^ | 0.03 (0.01-0.06) | 1.77 X 10^-02^ | -0.03 (-0.05-0.00) | 6.85 X 10^-02^ | 0.03 (0.01-0.06) | 7.62 X 10^-03^ |
| XLHDLFCp | 0.00 (-0.02-0.03) | 9.07 X 10^-01^ | 0.02 (-0.01-0.04) | 2.41 X 10^-01^ | 0.00 (-0.02-0.03) | 9.35 X 10^-01^ | 0.02 (0.00-0.05) | 1.09 X 10^-01^ |
| XLHDLTGp | -0.05 (-0.07--0.02) | 2.53 X 10^-04^ | 0.07 (0.05-0.10) | 1.19 X 10^-07^ | -0.04 (-0.06--0.01) | 7.72 X 10^-03^ | 0.07 (0.04-0.09) | 6.49 X 10^-07^ |
| LHDLPLp | -0.05 (-0.07--0.03) | 6.66 X 10^-05^ | 0.03 (0.00-0.05) | 4.17 X 10^-02^ | -0.05 (-0.07--0.02) | 7.22 X 10^-04^ | 0.02 (0.00-0.04) | 1.60 X 10^-01^ |
| LHDLCp | 0.05 (0.03-0.08) | 3.06 X 10^-05^ | -0.04 (-0.06--0.02) | 2.00 X 10^-03^ | 0.05 (0.02-0.07) | 5.89 X 10^-04^ | -0.03 (-0.06--0.01) | 9.34 X 10^-03^ |
| LHDLCEp | 0.05 (0.03-0.07) | 9.96 X 10^-05^ | -0.04 (-0.06--0.01) | 7.28 X 10^-03^ | 0.04 (0.02-0.07) | 1.19 X 10^-03^ | -0.03 (-0.05--0.01) | 2.44 X 10^-02^ |
| LHDLFCp | 0.06 (0.03-0.08) | 1.57 X 10^-05^ | -0.05 (-0.07--0.02) | 3.15 X 10^-04^ | 0.05 (0.03-0.07) | 4.54 X 10^-04^ | -0.04 (-0.06--0.02) | 1.97 X 10^-03^ |
| LHDLTGp | -0.04 (-0.07--0.02) | 1.44 X 10^-03^ | 0.04 (0.02-0.07) | 1.67 X 10^-03^ | -0.04 (-0.06--0.01) | 6.58 X 10^-03^ | 0.05 (0.02-0.07) | 7.13 X 10^-04^ |
| MHDLPLp | -0.04 (-0.07--0.02) | 3.26 X 10^-03^ | 0.03 (0.01-0.06) | 3.04 X 10^-02^ | -0.03 (-0.06--0.01) | 1.58 X 10^-02^ | 0.03 (0.01-0.06) | 2.85 X 10^-02^ |
| MHDLCp | 0.06 (0.04-0.09) | 1.95 X 10^-06^ | -0.05 (-0.08--0.03) | 1.85 X 10^-04^ | 0.05 (0.03-0.08) | 2.98 X 10^-04^ | -0.05 (-0.07--0.02) | 5.62 X 10^-04^ |
| MHDLCEp | 0.06 (0.03-0.08) | 2.73 X 10^-05^ | -0.05 (-0.08--0.03) | 1.85 X 10^-04^ | 0.05 (0.02-0.07) | 1.17 X 10^-03^ | -0.05 (-0.07--0.02) | 3.80 X 10^-04^ |
| MHDLFCp | 0.05 (0.03-0.08) | 8.18 X 10^-06^ | -0.03 (-0.05--0.01) | 2.77 X 10^-02^ | 0.04 (0.02-0.07) | 7.00 X 10^-04^ | -0.02 (-0.04-0.00) | 9.38 X 10^-02^ |
| MHDLTGp | -0.07 (-0.09--0.04) | 2.90 X 10^-07^ | 0.05 (0.03-0.08) | 1.06 X 10^-04^ | -0.06 (-0.08--0.03) | 1.96 X 10^-04^ | 0.05 (0.02-0.07) | 5.54 X 10^-04^ |
| SHDLPLp | -0.04 (-0.07--0.02) | 1.62 X 10^-03^ | 0.05 (0.03-0.08) | 7.03 X 10^-05^ | -0.03 (-0.06--0.01) | 2.65 X 10^-02^ | 0.04 (0.02-0.07) | 2.00 X 10^-03^ |
| SHDLCp | 0.06 (0.03-0.08) | 1.37 X 10^-05^ | -0.07 (-0.10--0.05) | 1.92 X 10^-07^ | 0.04 (0.02-0.07) | 1.78 X 10^-03^ | -0.06 (-0.08--0.03) | 2.38 X 10^-05^ |
| SHDLCEp | 0.06 (0.03-0.08) | 2.80 X 10^-05^ | -0.07 (-0.10--0.05) | 1.29 X 10^-07^ | 0.04 (0.02-0.07) | 3.16 X 10^-03^ | -0.06 (-0.08--0.03) | 1.60 X 10^-05^ |
| SHDLFCp | -0.03 (-0.05-0.00) | 4.47 X 10^-02^ | 0.06 (0.03-0.08) | 2.25 X 10^-05^ | -0.02 (-0.04-0.01) | 3.07 X 10^-01^ | 0.04 (0.02-0.07) | 1.12 X 10^-03^ |
| SHDLTGp | -0.07 (-0.09--0.05) | 2.09 X 10^-07^ | 0.06 (0.03-0.08) | 7.59 X 10^-06^ | -0.06 (-0.08--0.03) | 1.96 X 10^-04^ | 0.05 (0.03-0.08) | 1.22 X 10^-04^ |
| VLDLD | -0.08 (-0.10--0.05) | 1.68 X 10^-08^ | 0.08 (0.06-0.10) | 5.84 X 10^-10^ | -0.06 (-0.08--0.04) | 4.79 X 10^-05^ | 0.07 (0.04-0.09) | 1.23 X 10^-07^ |
| LDLD | -0.01 (-0.03-0.02) | 5.31 X 10^-01^ | 0.01 (-0.02-0.03) | 6.72 X 10^-01^ | -0.01 (-0.04-0.01) | 5.11 X 10^-01^ | 0.01 (-0.01-0.03) | 4.75 X 10^-01^ |
| HDLD | 0.05 (0.03-0.07) | 6.79 X 10^-06^ | -0.02 (-0.04-0.00) | 7.33 X 10^-02^ | 0.04 (0.02-0.06) | 5.89 X 10^-04^ | -0.01 (-0.03-0.01) | 3.43 X 10^-01^ |
| SerumC | 0.02 (-0.01-0.04) | 2.51 X 10^-01^ | 0.02 (-0.01-0.04) | 1.98 X 10^-01^ | 0.01 (-0.01-0.03) | 4.89 X 10^-01^ | 0.03 (0.01-0.06) | 8.13 X 10^-03^ |
| VLDLC | -0.06 (-0.08--0.03) | 2.73 X 10^-05^ | 0.07 (0.04-0.09) | 3.94 X 10^-07^ | -0.05 (-0.07--0.02) | 1.07 X 10^-03^ | 0.07 (0.04-0.09) | 3.23 X 10^-07^ |
| RemnantC | -0.03 (-0.06--0.01) | 1.74 X 10^-02^ | 0.05 (0.03-0.08) | 1.29 X 10^-04^ | -0.03 (-0.05-0.00) | 4.10 X 10^-02^ | 0.06 (0.04-0.08) | 5.01 X 10^-06^ |
| LDLC | 0.02 (-0.01-0.04) | 2.05 X 10^-01^ | 0.00 (-0.02-0.03) | 9.28 X 10^-01^ | 0.01 (-0.01-0.03) | 4.86 X 10^-01^ | 0.02 (-0.01-0.04) | 1.95 X 10^-01^ |
| HDLC | 0.06 (0.03-0.08) | 2.07 X 10^-06^ | -0.02 (-0.04-0.00) | 7.14 X 10^-02^ | 0.04 (0.02-0.07) | 3.92 X 10^-04^ | -0.01 (-0.04-0.01) | 2.66 X 10^-01^ |
| HDL2C | 0.05 (0.03-0.08) | 2.97 X 10^-06^ | -0.02 (-0.04-0.00) | 5.82 X 10^-02^ | 0.04 (0.02-0.07) | 4.54 X 10^-04^ | -0.01 (-0.04-0.01) | 2.26 X 10^-01^ |
| HDL3C | 0.05 (0.03-0.08) | 1.19 X 10^-05^ | -0.01 (-0.03-0.01) | 4.91 X 10^-01^ | 0.05 (0.02-0.07) | 4.07 X 10^-04^ | 0.00 (-0.02-0.02) | 9.17 X 10^-01^ |
| EstC | 0.02 (0.00-0.04) | 1.51 X 10^-01^ | 0.02 (-0.01-0.04) | 2.15 X 10^-01^ | 0.01 (-0.01-0.04) | 3.42 X 10^-01^ | 0.03 (0.01-0.06) | 1.04 X 10^-02^ |
| FreeC | 0.01 (-0.02-0.03) | 6.69 X 10^-01^ | 0.02 (-0.01-0.04) | 1.77 X 10^-01^ | 0.00 (-0.02-0.02) | 9.35 X 10^-01^ | 0.03 (0.01-0.06) | 7.04 X 10^-03^ |
| SerumTG | -0.07 (-0.09--0.05) | 2.29 X 10^-07^ | 0.08 (0.06-0.11) | 4.60 X 10^-10^ | -0.05 (-0.08--0.03) | 2.14 X 10^-04^ | 0.08 (0.05-0.10) | 1.40 X 10^-08^ |
| VLDLTG | -0.07 (-0.09--0.05) | 2.09 X 10^-07^ | 0.08 (0.06-0.11) | 3.96 X 10^-10^ | -0.05 (-0.08--0.03) | 1.96 X 10^-04^ | 0.07 (0.05-0.10) | 1.63 X 10^-08^ |
| LDLTG | -0.02 (-0.04-0.00) | 1.40 X 10^-01^ | 0.05 (0.02-0.07) | 5.86 X 10^-04^ | -0.01 (-0.04-0.01) | 3.40 X 10^-01^ | 0.05 (0.03-0.07) | 1.76 X 10^-04^ |
| HDLTG | -0.05 (-0.08--0.03) | 7.66 X 10^-05^ | 0.08 (0.05-0.10) | 6.35 X 10^-09^ | -0.04 (-0.07--0.02) | 4.12 X 10^-03^ | 0.08 (0.05-0.10) | 3.20 X 10^-08^ |
| DAG | -0.06 (-0.09--0.04) | 1.38 X 10^-05^ | 0.03 (0.01-0.06) | 2.13 X 10^-02^ | -0.05 (-0.08--0.03) | 6.17 X 10^-04^ | 0.03 (0.00-0.06) | 4.81 X 10^-02^ |
| DAGTG | -0.03 (-0.05-0.00) | 9.22 X 10^-02^ | -0.01 (-0.04-0.02) | 5.23 X 10^-01^ | -0.02 (-0.05-0.00) | 1.55 X 10^-01^ | -0.01 (-0.04-0.02) | 4.56 X 10^-01^ |
| TotPG | -0.01 (-0.03-0.01) | 4.63 X 10^-01^ | 0.05 (0.03-0.07) | 1.24 X 10^-04^ | -0.01 (-0.03-0.02) | 6.03 X 10^-01^ | 0.05 (0.03-0.08) | 1.72 X 10^-05^ |
| TGPG | -0.07 (-0.10--0.05) | 3.75 X 10^-08^ | 0.08 (0.05-0.10) | 5.07 X 10^-09^ | -0.06 (-0.08--0.03) | 1.16 X 10^-04^ | 0.06 (0.04-0.09) | 7.91 X 10^-07^ |
| PC | 0.00 (-0.03-0.02) | 7.37 X 10^-01^ | 0.05 (0.03-0.08) | 3.32 X 10^-05^ | 0.00 (-0.03-0.02) | 8.70 X 10^-01^ | 0.06 (0.04-0.08) | 2.63 X 10^-06^ |
| SM | 0.00 (-0.02-0.02) | 9.37 X 10^-01^ | 0.00 (-0.03-0.02) | 7.29 X 10^-01^ | -0.01 (-0.03-0.02) | 7.10 X 10^-01^ | 0.01 (-0.02-0.03) | 6.12 X 10^-01^ |
| TotCho | -0.01 (-0.03-0.01) | 4.88 X 10^-01^ | 0.03 (0.00-0.05) | 3.17 X 10^-02^ | -0.01 (-0.03-0.02) | 5.58 X 10^-01^ | 0.03 (0.01-0.06) | 9.38 X 10^-03^ |
| ApoA1 | 0.04 (0.02-0.06) | 7.91 X 10^-04^ | 0.01 (-0.01-0.03) | 4.27 X 10^-01^ | 0.03 (0.01-0.05) | 6.98 X 10^-03^ | 0.02 (0.00-0.04) | 1.06 X 10^-01^ |
| ApoB | -0.03 (-0.06--0.01) | 2.03 X 10^-02^ | 0.05 (0.03-0.08) | 7.60 X 10^-05^ | -0.03 (-0.05-0.00) | 5.83 X 10^-02^ | 0.06 (0.04-0.09) | 4.41 X 10^-06^ |
| ApoBApoA1 | -0.05 (-0.07--0.03) | 7.66 X 10^-05^ | 0.05 (0.02-0.07) | 2.52 X 10^-04^ | -0.04 (-0.07--0.02) | 1.32 X 10^-03^ | 0.05 (0.03-0.07) | 1.28 X 10^-04^ |
| TotFA | -0.04 (-0.06--0.01) | 4.42 X 10^-03^ | 0.08 (0.05-0.10) | 1.64 X 10^-08^ | -0.03 (-0.05--0.01) | 3.70 X 10^-02^ | 0.08 (0.05-0.10) | 1.11 X 10^-08^ |
| FALen | -0.04 (-0.07--0.02) | 2.26 X 10^-03^ | 0.03 (0.00-0.05) | 7.14 X 10^-02^ | -0.03 (-0.06--0.01) | 2.97 X 10^-02^ | 0.01 (-0.01-0.04) | 4.08 X 10^-01^ |
| UnSat | 0.05 (0.03-0.08) | 8.07 X 10^-05^ | -0.07 (-0.09--0.04) | 3.07 X 10^-07^ | 0.04 (0.02-0.07) | 3.85 X 10^-03^ | -0.07 (-0.09--0.04) | 7.88 X 10^-07^ |
| DHA | 0.01 (-0.01-0.04) | 3.37 X 10^-01^ | 0.01 (-0.01-0.04) | 4.87 X 10^-01^ | 0.01 (-0.01-0.04) | 4.30 X 10^-01^ | 0.01 (-0.01-0.04) | 4.28 X 10^-01^ |
| LA | -0.01 (-0.04-0.01) | 3.65 X 10^-01^ | 0.03 (0.01-0.06) | 2.12 X 10^-02^ | -0.02 (-0.04-0.01) | 2.40 X 10^-01^ | 0.04 (0.02-0.07) | 6.68 X 10^-04^ |
| CLA | -0.04 (-0.07--0.01) | 4.51 X 10^-03^ | 0.04 (0.02-0.07) | 3.74 X 10^-03^ | -0.04 (-0.06--0.01) | 1.49 X 10^-02^ | 0.04 (0.02-0.07) | 3.33 X 10^-03^ |
| FAw3 | 0.01 (-0.02-0.03) | 5.86 X 10^-01^ | 0.03 (0.00-0.05) | 7.14 X 10^-02^ | 0.01 (-0.02-0.03) | 5.49 X 10^-01^ | 0.03 (0.00-0.05) | 4.82 X 10^-02^ |
| FAw6 | -0.01 (-0.04-0.01) | 3.51 X 10^-01^ | 0.04 (0.01-0.06) | 5.11 X 10^-03^ | -0.01 (-0.04-0.01) | 3.39 X 10^-01^ | 0.05 (0.02-0.07) | 3.45 X 10^-04^ |
| PUFA | -0.01 (-0.04-0.01) | 4.62 X 10^-01^ | 0.04 (0.01-0.06) | 5.78 X 10^-03^ | -0.01 (-0.04-0.01) | 4.57 X 10^-01^ | 0.05 (0.02-0.07) | 4.94 X 10^-04^ |
| MUFA | -0.05 (-0.08--0.03) | 1.32 X 10^-04^ | 0.08 (0.06-0.11) | 7.27 X 10^-10^ | -0.04 (-0.06--0.01) | 8.07 X 10^-03^ | 0.08 (0.05-0.10) | 8.47 X 10^-09^ |
| SFA | -0.04 (-0.06--0.01) | 3.46 X 10^-03^ | 0.08 (0.06-0.11) | 7.27 X 10^-10^ | -0.03 (-0.05-0.00) | 4.19 X 10^-02^ | 0.08 (0.06-0.11) | 1.38 X 10^-09^ |
| DHAFA | 0.05 (0.02-0.07) | 4.18 X 10^-04^ | -0.04 (-0.06--0.01) | 8.14 X 10^-03^ | 0.04 (0.01-0.06) | 5.47 X 10^-03^ | -0.04 (-0.06--0.01) | 9.38 X 10^-03^ |
| LAFA | 0.04 (0.02-0.07) | 9.62 X 10^-04^ | -0.07 (-0.09--0.04) | 1.09 X 10^-07^ | 0.02 (0.00-0.04) | 1.05 X 10^-01^ | -0.05 (-0.07--0.03) | 4.24 X 10^-05^ |
| CLAFA | -0.03 (-0.05-0.00) | 8.81 X 10^-02^ | 0.02 (-0.01-0.04) | 2.75 X 10^-01^ | -0.02 (-0.05-0.00) | 1.30 X 10^-01^ | 0.02 (-0.01-0.04) | 2.53 X 10^-01^ |
| FAw3FA | 0.05 (0.03-0.08) | 7.53 X 10^-05^ | -0.04 (-0.06--0.01) | 9.93 X 10^-03^ | 0.05 (0.02-0.07) | 1.04 X 10^-03^ | -0.03 (-0.06--0.01) | 1.39 X 10^-02^ |
| FAw6FA | 0.05 (0.03-0.08) | 2.26 X 10^-05^ | -0.08 (-0.11--0.06) | 1.70 X 10^-10^ | 0.03 (0.01-0.06) | 9.42 X 10^-03^ | -0.07 (-0.09--0.04) | 6.66 X 10^-08^ |
| PUFAFA | 0.06 (0.04-0.09) | 2.25 X 10^-06^ | -0.09 (-0.11--0.06) | 6.16 X 10^-11^ | 0.04 (0.02-0.07) | 2.00 X 10^-03^ | -0.07 (-0.10--0.05) | 2.23 X 10^-08^ |
| MUFAFA | -0.05 (-0.08--0.03) | 8.07 X 10^-05^ | 0.06 (0.03-0.08) | 9.06 X 10^-06^ | -0.04 (-0.06--0.01) | 9.66 X 10^-03^ | 0.05 (0.02-0.07) | 3.54 X 10^-04^ |
| SFAFA | -0.02 (-0.04-0.01) | 2.17 X 10^-01^ | 0.05 (0.02-0.07) | 7.24 X 10^-04^ | -0.01 (-0.04-0.01) | 4.89 X 10^-01^ | 0.04 (0.02-0.07) | 1.97 X 10^-03^ |
| Glc | -0.05 (-0.08--0.03) | 3.48 X 10^-05^ | 0.04 (0.02-0.07) | 8.46 X 10^-04^ | -0.04 (-0.07--0.02) | 1.54 X 10^-03^ | 0.03 (0.01-0.06) | 1.64 X 10^-02^ |
| Lac | -0.07 (-0.10--0.05) | 2.29 X 10^-07^ | 0.08 (0.06-0.11) | 1.28 X 10^-09^ | -0.06 (-0.09--0.04) | 4.79 X 10^-05^ | 0.07 (0.05-0.10) | 4.40 X 10^-08^ |
| Cit | 0.00 (-0.02-0.03) | 7.93 X 10^-01^ | -0.04 (-0.07--0.02) | 1.12 X 10^-03^ | 0.00 (-0.02-0.03) | 9.09 X 10^-01^ | -0.04 (-0.07--0.02) | 1.20 X 10^-03^ |
| Ala | -0.04 (-0.07--0.02) | 2.76 X 10^-03^ | 0.08 (0.06-0.11) | 2.17 X 10^-09^ | -0.03 (-0.06--0.01) | 2.96 X 10^-02^ | 0.07 (0.05-0.10) | 7.54 X 10^-08^ |
| Gln | 0.03 (0.00-0.05) | 5.46 X 10^-02^ | -0.04 (-0.07--0.02) | 1.11 X 10^-03^ | 0.02 (0.00-0.05) | 1.54 X 10^-01^ | -0.04 (-0.06--0.02) | 3.03 X 10^-03^ |
| His | 0.02 (-0.01-0.04) | 2.80 X 10^-01^ | 0.01 (-0.01-0.04) | 3.88 X 10^-01^ | 0.01 (-0.01-0.04) | 3.75 X 10^-01^ | 0.02 (-0.01-0.04) | 2.87 X 10^-01^ |
| Ile | -0.08 (-0.10--0.05) | 2.81 X 10^-09^ | 0.07 (0.05-0.10) | 7.27 X 10^-10^ | -0.06 (-0.08--0.04) | 7.79 X 10^-06^ | 0.07 (0.04-0.09) | 6.51 X 10^-08^ |
| Leu | -0.06 (-0.08--0.04) | 2.29 X 10^-07^ | 0.07 (0.05-0.10) | 1.70 X 10^-10^ | -0.05 (-0.07--0.03) | 1.62 X 10^-04^ | 0.07 (0.04-0.09) | 1.60 X 10^-08^ |
| Val | -0.04 (-0.06--0.01) | 2.88 X 10^-03^ | 0.04 (0.02-0.06) | 1.48 X 10^-03^ | -0.03 (-0.06--0.01) | 9.27 X 10^-03^ | 0.03 (0.01-0.06) | 5.77 X 10^-03^ |
| Phe | -0.06 (-0.08--0.03) | 2.02 X 10^-05^ | 0.05 (0.03-0.07) | 1.85 X 10^-04^ | -0.05 (-0.07--0.02) | 7.00 X 10^-04^ | 0.04 (0.02-0.07) | 9.49 X 10^-04^ |
| Tyr | -0.04 (-0.07--0.02) | 1.02 X 10^-03^ | 0.05 (0.02-0.07) | 8.32 X 10^-04^ | -0.04 (-0.06--0.01) | 6.58 X 10^-03^ | 0.04 (0.02-0.07) | 2.04 X 10^-03^ |
| Ace | 0.03 (0.01-0.06) | 1.31 X 10^-02^ | -0.05 (-0.08--0.03) | 8.36 X 10^-05^ | 0.03 (0.00-0.05) | 4.53 X 10^-02^ | -0.05 (-0.07--0.02) | 4.02 X 10^-04^ |
| bOHBut | -0.01 (-0.04-0.01) | 4.53 X 10^-01^ | -0.03 (-0.05-0.00) | 3.93 X 10^-02^ | -0.01 (-0.04-0.01) | 4.50 X 10^-01^ | -0.03 (-0.06--0.01) | 2.98 X 10^-02^ |
| Crea | 0.00 (-0.02-0.03) | 7.30 X 10^-01^ | 0.03 (0.01-0.05) | 4.82 X 10^-03^ | 0.01 (-0.01-0.03) | 6.27 X 10^-01^ | 0.03 (0.01-0.05) | 6.21 X 10^-03^ |
| Alb | 0.01 (-0.01-0.04) | 4.41 X 10^-01^ | 0.00 (-0.02-0.03) | 8.79 X 10^-01^ | 0.00 (-0.02-0.03) | 8.53 X 10^-01^ | 0.00 (-0.02-0.03) | 8.26 X 10^-01^ |
| Gp | -0.10 (-0.13--0.08) | 1.73 X 10^-13^ | 0.08 (0.05-0.10) | 2.20 X 10^-09^ | -0.08 (-0.11--0.06) | 1.42 X 10^-08^ | 0.07 (0.04-0.09) | 2.37 X 10^-07^ |

Model 1: adjusted for age, sex, and education. Model 2 adjusted for, age, sex, education, smoking, alcohol consumption, caloric intake, ethnicity and physical activity. Model 3 adjusted for, age, sex, education, smoking, alcohol consumption, caloric intake, ethnicity, physical activity, lipid lowering drugs, and antidepressants. FDR significant (q < 0.05)

# **Supplemental table 3.** The linear regression analysis of the association between the sum score of the symptoms extracted from the metabolite-symptom clustering step and the cardiometabolic diseases.

|  | Crude | | Model 1 | | Model 2 | | Model 3 | |
| --- | --- | --- | --- | --- | --- | --- | --- | --- |
|  | Overall Depression | IMD | Overall Depression | IMD | Overall Depression | IMD | Overall Depression | IMD |
| Cardiometabolic diseases | β (95% CI) | β (95% CI) | β (95% CI) | β (95% CI) | β (95% CI) | β (95% CI) | β (95% CI) | β (95% CI) |
| BMI (kg/m^2^) | -0.11  (-0.14--0.09)* | 0.10  (0.07-0.12)* | -0.11  (-0.13--0.08)* | 0.08  (0.06-0.11)* | -0.10  (-0.12--0.07)* | 0.08  (0.05-0.10)* | -0.08  (-0.11--0.06)* | 0.07  (0.05-0.09)* |
| Total body fat (%) | -0.05  (-0.08--0.03)* | 0.23  (0.20-0.25)* | -0.07  (-0.09--0.06)* | 0.06  (0.05-0.08)* | -0.07  (-0.09--0.05)* | 0.06  (0.05-0.08)* | -0.06  (-0.08--0.04)* | 0.06  (0.04-0.07)* |
| Waist circumference (cm) | -0.12  (-0.14--0.09)* | 0.03  (0.01-0.06)* | -0.10  (-0.13--0.08)* | 0.09  (0.07-0.11)* | -0.10  (-0.12--0.07)* | 0.09  (0.06-0.11)* | -0.08  (-0.10--0.06)* | 0.07  (0.05-0.10)* |
| Visceral adipose tissue (cm^2^)* | -0.12  (-0.16--0.08)* | 0.02  (-0.02-0.06) | -0.10  (-0.14--0.07)* | 0.08  (0.04-0.12)* | -0.09  (-0.13--0.06)* | 0.07  (0.03-0.11)* | -0.08  (-0.12--0.04)* | 0.05  (0.01-0.09)* |
| Fasting glucose (mmol/L) | -0.04  (-0.07--0.02)* | 0.05  (0.02-0.07)* | -0.05  (-0.08--0.03)* | 0.06  (0.04-0.08)* | -0.05  (-0.07--0.03)* | 0.06  (0.03-0.08)* | -0.04  (-0.06--0.01)* | 0.05  (0.02-0.07)* |
| HOMA-1B | -0.07  (-0.10--0.05)* | 0.05  (0.02-0.07)* | -0.06  (-0.09--0.04)* | 0.06  (0.04-0.09)* | -0.06  (-0.08--0.03)* | 0.06  (0.03-0.08)* | -0.06  (-0.08--0.03)* | 0.05  (0.03-0.08)* |
| HOMA-IR | -0.07  (-0.09--0.04)* | 0.06  (0.04-0.09)* | -0.07  (-0.09--0.05)* | 0.07  (0.05-0.10)* | -0.06  (-0.09--0.04)* | 0.07  (0.04-0.09)* | -0.05  (-0.08--0.03)* | 0.06  (0.03-0.09)* |
| HbA1c (%) | -0.06  (-0.09--0.04)* | 0.07  (0.05-0.10)* | -0.07  (-0.10--0.05)* | 0.07  (0.04-0.09)* | -0.06  (-0.09--0.04)* | 0.06  (0.04-0.09)* | -0.05  (-0.08--0.03)* | 0.05  (0.03-0.07)* |
| Total cholesterol (mmol/L) | 0.01  (-0.01-0.04) | 0.06  (0.03-0.08)* | 0.00  (-0.02-0.03) | 0.03  (0.00-0.05)* | 0.00  (-0.02-0.03) | 0.03  (0.00-0.05) | 0.00  (-0.03-0.02) | 0.04  (0.02-0.06)* |
| HDL-cholesterol (mmol/L) | 0.09  (0.07-0.11)* | 0.07  (0.04-0.09)* | 0.07  (0.05-0.09)* | -0.03  (-0.05--0.01)* | 0.05  (0.03-0.07)* | -0.02  (-0.04-0.00) | 0.04  (0.02-0.07)* | -0.01  (-0.04-0.01) |
| Triglycerides (mmol/L) | -0.09  (-0.11--0.07)* | 0.05  (0.02-0.07)* | -0.08  (-0.11--0.06)* | 0.09  (0.06-0.11)* | -0.07  (-0.10--0.05)* | 0.08  (0.06-0.11)* | -0.06  (-0.08--0.03)* | 0.08  (0.05-0.10)* |
| LDL-cholesterol (mmol/L) | 0.01  (-0.01-0.04) | 0.02  (-0.01-0.04) | 0.01  (-0.01-0.04) | 0.00  (-0.02-0.03) | 0.01  (-0.01-0.04) | 0.00  (-0.02-0.03) | 0.00  (-0.02-0.03) | 0.02  (-0.01-0.04) |

Number of individuals with data for BMI: 6572, Total body fat: 6541, Waist circumference: 6566, Visceral adipose tissue: 2537, Fasting glucose: 6554, HOMA-1B: 6541, HOMA-IR: 6545, HbA1c: 6543, Total cholesterol: 6562, HDL-cholesterol: 6561, Triglycerides: 6561, LDL-cholesterol: 6560. Model 1: adjusted for age, sex, and education. Model 2 adjusted for, age, sex, education, smoking, alcohol consumption, caloric intake, ethnicity and physical activity. Model 3 adjusted for, age, sex, education, smoking, alcohol consumption, caloric intake, ethnicity, physical activity, lipid lowering drugs, and antidepressants. * FDR significant (q < 0.05)

# **References**

Bot, M., Milaneschi, Y., Al-Shehri, T., Amin, N., Garmaeva, S., Onderwater, G. L. J., . . . Penninx, B. (2020). Metabolomics Profile in Depression: A Pooled Analysis of 230 Metabolic Markers in 5283 Cases With Depression and 10,145 Controls. *Biological Psychiatry, 87*(5), 409-418. doi:10.1016/j.biopsych.2019.08.016

Buch, A. M., & Liston, C. (2021). Dissecting diagnostic heterogeneity in depression by integrating neuroimaging and genetics. *Neuropsychopharmacology, 46*(1), 156-175. doi:10.1038/s41386-020-00789-3

Hotelling, H. (1936). Relations Between Two Sets of Variates. *Biometrika, 28*(3/4), 321-377. doi:10.2307/2333955

Jolliffe, I. T. (2002). *Springer Series in statistics. Principal Component Analysis* (Second Edition. ed.). New York: Springer-Verlag.

Onderwater, G. L. J., Ligthart, L., Bot, M., Demirkan, A., Fu, J., van der Kallen, C. J. H., . . . van den Maagdenberg, A. (2019). Large-scale plasma metabolome analysis reveals alterations in HDL metabolism in migraine. *Neurology, 92*(16), e1899-e1911. doi:10.1212/wnl.0000000000007313

Soininen, P., Kangas, A. J., Würtz, P., Suna, T., & Ala-Korpela, M. (2015). Quantitative serum nuclear magnetic resonance metabolomics in cardiovascular epidemiology and genetics. *Circulation. Cardiovascular Genetics, 8*(1), 192-206. doi:10.1161/circgenetics.114.000216
